# Mechanochemical synthesis of Knoevenagel condensation products from biorenewable furaldehydes using crustacean waste-derived chitosan as a sustainable organocatalyst†

**DOI:** 10.1039/d5ra02836a

**Published:** 2025-06-10

**Authors:** Rachitha S N, Abhishek Kumar Yadav, Mangalapalli Kamali, Putla Sudarsanam, Saikat Dutta

**Affiliations:** a Department of Chemistry, National Institute of Technology Karnataka (NITK) Surathkal Mangalore 575025 India sdutta@nitk.edu.in; b Department of Chemistry, Indian Institute of Technology Hyderabad Kandi 502284 Telangana India

## Abstract

The biorefinery processes employing renewable feedstock can benefit from sustainable synthetic practices, such as mechanochemistry, organocatalysis, and renewable catalysts. This work reports using crustacean waste-derived chitosan (CS) as an eco-friendly and recyclable heterogeneous organocatalyst for the Knoevenagel condensation reaction between biorenewable 5-substituted-2-furaldehydes and malononitrile. The reaction was performed under solvent-free, mechanochemical conditions in a mortar and pestle. The reaction kinetics were faster, and the product selectivity was higher under mechanochemical conditions than in solvent-mediated synthesis. The CS catalyst was conveniently recovered and recycled. Moreover, the Knoevenagel condensation reaction was extended to substituted benzaldehydes to demonstrate the broad substrate scope of the process. In all cases, the Knoevenagel condensation products were isolated in excellent yields (>85%) in <30 min at RT. The CS (fresh and recycled) catalysts were characterized by UV-Vis, FTIR, PXRD, SEM-EDX, DSC, TGA, and elemental analysis techniques.

## Abstract 2

The Knoevenagel condensation reaction between biomass-derived furaldehydes and malononitrile afforded excellent isolated yields of the products at ambient temperature under solvent-free mechanochemistry using chitosan as a recyclable organocatalyst.

## Graphical abstract

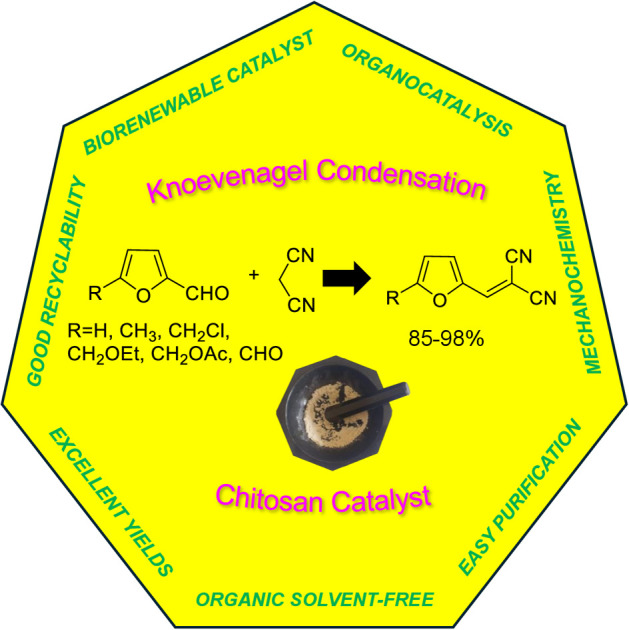

## Introduction

Catalysis is ingrained in sustainable synthesis, lowering the activation energy of chemical transformations, increasing the selectivity of targeted products by favoring a specific mechanistic pathway, allowing the use of easily available and innocuous starting materials, and reducing waste generation.^1^ Major industrial applications of catalysts include transforming petroleum-derived hydrocarbons into functionalized organic molecules and synthetic polymers.^2^ Current research is focused on developing increasingly more efficient catalysts in terms of activity, substrate scope, selectivity, non-toxicity, and recyclability to maximize the efficiency of chemical transformations involving petrochemicals.^3^ However, since petroleum is an exhaustive, anthropogenic carbon-based feedstock, even the most efficient catalytic transformations of petrochemicals cannot make the organic chemical industry sustainable in the true sense. Significant interest has been given to abundant terrestrial and marine biomass, especially those considered wastes, as renewable carbon-based feedstock that can reduce the dependency on petroleum for synthesizing organic chemicals.^4^ There is significant interest in sourcing catalysts from biomass for a closed-loop biorefinery approach since they are non-toxic, biodegradable, and mitigate waste management issues.^5^ In this regard, crustacean exoskeletons (*e.g.*, shrimp and crab) produced in the seafood processing industry are the major waste materials. The enormous production of this waste outpaces its prevalent applications and occasionally leads to ecological disasters in the coastal region due to improper disposal.^6^ The three major chemical constituents of this waste are protein, calcite, and chitin. Chitin, separated from crustacean waste by deproteinization and demineralization steps, is a linear biopolymer made of *N*-acetylglucosamine. Chitosan (CS), routinely produced by the base-promoted deacetylation of chitin, has been used as a sustainable catalyst in various organic transformations.^7^ Among the various classes of catalysts, organocatalysts have received particular attention since they are stable organic molecules, available in high purity, non-toxic, and selective.^8^ Moreover, the reactions using organocatalysts work under relatively mild conditions and do not produce waste containing metal salts. Interestingly, CS has been employed as a heterogeneous and biorenewable organocatalyst for organic transformations.^9,10^ As an innocuous, renewable, and biodegradable catalyst, CS has been used as a sustainable and green catalyst in various organic transformations.^11^

Carbohydrates, including chitin, are major components in terrestrial and aquatic biomass. A chemical-catalytic pathway of the value-addition of carbohydrates involves their hydrolysis and dehydration into furanics and levulinic acid platform chemicals.^12^ The platform chemicals are then synthetically transformed into chemicals and materials of desired structure, properties, and applications. Furfural (FF, 1a) is produced by the dehydration of pentose sugars (*e.g.*, xylose) in the hemicellulose fraction of lignocellulosic biomass.^13^ 5-(Hydroxymethyl)furfural (HMF, 1b) is a well-documented platform chemical sourced from hexose sugars (*e.g.*, glucose) and polymeric carbohydrates (*e.g.*, cellulose). As carbohydrate-derived platform chemicals, 1a and 1b are at the forefront of biorefinery research. The hydroxymethyl group in HMF can be altered synthetically to produce 5-substituted-2-furaldehydes.^14^ Hydrogenolysis of the hydroxymethyl group in HMF leads to 5-methylfurfural (MF, 1c). Converting the hydroxymethyl group in HMF into an ethyl ether leads to 5-(ethoxymethyl)furfural (EMF, 1d), whereas the esterification of HMF with acetic acid leads to 5-(acetoxymethyl)furfural (AcMF, 1f). The reaction of a molecule of HMF with HCl leads to 5-(chloromethyl)furfural (CMF, 1e). Oxidation of the hydroxymethyl group in HMF leads to 2,5-diformylfuran (DFF, 1g).

Even though organic reactions are routinely performed using a liquid medium (*e.g.*, organic solvent), serious considerations have been given to performing them in solvent-free conditions owing to the specific advantages of the latter approach. In mechanochemistry, a chemical reaction proceeds by bringing the reactants close at the molecular level using mechanical energy.^15^ Organic synthesis using mechanochemistry can be performed using a mortar and pestle or automated ball mills.^16^ Mechanochemistry has many advantages over conventional processes that require bulk dissolution of reactants in organic solvents. For example, mechanochemical synthesis can be performed under solvent-free conditions, eliminating up to 90% of the reaction mass. Moreover, the mechanochemical processes often have faster kinetics than solution phase synthesis, minimize energy consumption, improve product selectivity and yield, simplify product purification, and often work under ambient conditions.^15,17^ Therefore, mechanochemical synthesis qualifies as green chemistry. The biopolymers in biomass can be transformed into tailored chemicals as well as materials using mechanochemistry.^18,19^ Interestingly, several works reported the base-promoted preparation of CS from chitin and crustacean shells under solvent-free or solvent-assisted conditions using ball-milling or a mortar and a pestle.^20–22^ Even though the scope of mechanochemistry for synthesizing small organic molecules has expanded rapidly over the past decade, its applications in the value-addition of biomass-derived platform chemicals remain underexplored.^23^

**Fig. 1:**
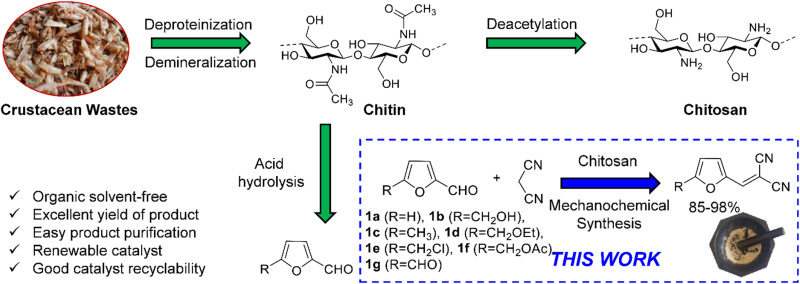
Mechanochemical synthesis of Knoevenagel condensation products by reacting biorenewable furaldehydes with malononitrile using CS as a heterogeneous organocatalyst.

Knoevenagel condensation reaction between aromatic aldehydes (*e.g.*, benzaldehyde) and active methylene compounds (*e.g.*, malononitrile) produces attractive molecules like benzylidenemalononitrile, which are useful as chemical intermediate for synthesizing targeted bioactive molecules with pharmacological properties.^24^ This elegant transformation typically works under mild conditions and produces water as the sole byproduct. This work reports on the mechanochemical synthesis of Knoevenagel condensation products starting from carbohydrate-derived 5-substituted-2-furaldehydes and malononitrile under organic solvent-free conditions using CS as a sustainable organocatalyst (Fig. 1). Knoevenagel condensation products from substituted benzaldehydes were also synthesized to demonstrate the broad substrate scope of the synthetic strategy.

## Experimental section

### Materials

Furfural (99%) was purchased from Spectrochem Pvt., Ltd. Malononitrile (98%) was purchased from Sisco Research Laboratories Pvt., Ltd. 5-Methylfurfural (MF, 99%) was purchased from Sigma. Ethyl acetate (99%) was purchased from Finar Limited. Sodium hydroxide (97%) was procured from Spectrochem Pvt., Ltd. 5-(Hydroxymethyl)furfural (HMF), 5-(chloromethyl)furfural (CMF), 5-(acetoxymethyl)furfural (AcMF), 5-(ethoxymethyl)furfural (EMF), and 2,5-diformylfuran (DFF) were synthesized and purified by following a literature procedure.^25–28^ Furfural was distilled and refrigerated in an airtight, amber-colored glass container. All other chemicals were used as received. Benzaldehyde (98%) was purchased from Spectrochem Pvt., Ltd. Anisaldehyde (98%) and 4-chlorobenzaldehyde (97%) were purchased from TCI Chemicals. 4-Ethoxybenzaldehyde (99%) was purchased from Sigma-Aldrich. Thin-layer chromatography (TLC) plates and silica gel pre-coated on aluminum sheets were purchased from Merck (TLC Silica Gel 60, F254). Chitin was procured from Otto Chemie Pvt. Ltd.

### Instrumentation

The synthesized Knoevenagel condensation products were characterized by melting point analysis and spectroscopic techniques. The Fourier transform infrared (FTIR) spectra of the organic compounds were collected in a Bruker Alpha II FTIR 400 instrument using the attenuated total reflectance (ATR) technique. The ^1^H NMR of the synthesized compounds was recorded using Bruker NanoBay® instrument operating at the frequency of 400 MHz. The ^13^C NMR spectra were acquired in the same instrument at calculated frequency of 100 MHz. The FTIR spectra of materials (chitin and CS) were collected using the same instrument by pelletizing them with anhydrous KBr. The crystallinity of the samples was analyzed by a powder X-ray diffractometer (PXRD) in an Empyrean 3rd Gen, Malvern PANalytical instrument using the Bragg's diffraction angles (2*θ*). Thermogravimetry analysis (TGA) was performed using the SDT Q600 TA instrument in the temperature range of 50–800 °C under the N_2_ atmosphere at the temperature ramp of 10 °C min^−1^. The differential scanning calorimetry was done using the TA Q200 instrument. The field-emission scanning electron microscopy (FE-SEM) images were collected using the JEOL JIB4700F FIB-SEM instrument. Samples were prepared by spin-coating the solutions onto a silicon substrate and dried under vacuum at room temperature. Samples were sputtered with gold for FE-SEM measurements. Elemental mapping of carbon, hydrogen, and oxygen was done using a JEOL JIB4700F FIB-SEM instrument equipped with energy-dispersive X-ray spectroscopy (EDS). UV-Visible spectra were recorded in a PerkinElmer (Lambda 950) spectrophotometer instrument.

### Preparation of catalyst

In the deacetylation experiment, chitin (3.00 g) was suspended in 45% NaOH (40 mL) taken in a round-bottomed flask. A stirring rod was introduced, and the suspension was placed in a preheated oil bath. A reflux condenser was attached to the flask, and the suspension was magnetically stirred at 120 °C (oil-bath temperature) for 72 h. After the reaction, the mixture was cooled to room temperature, diluted with water (100 mL), and filtered through a filter paper (Whatman, grade 5) under vacuum. The beige solid was washed with deionized water till the washing was neutral. Finally, the solid (*i.e.*, CS) was dried in a hot-air oven (80 °C, 6 h).

### Typical synthesis of Knoevenagel condensation products (3a–3j)

Furfural (0.500 g, 5.21 mmol) and malononitrile (0.344 g, 5.21 mmol) were taken in a clean and dried mortar. CS (0.050 g, 10 wt%) was added to the mixture, and the heterogeneous mixture was ground with the pestle. The mixture became thicker within a few minutes, indicating the formation of a solid product, and then the entire mass solidified into a beige powder. The reaction progress was monitored by thin-layer chromatography (TLC) for the disappearance of furfural. The solid powdered mass was dissolved in ethyl acetate (10 mL) and filtered through a filter paper (Whatman, Grade 5) under vacuum. The CS catalyst on the filter paper was washed with fresh ethyl acetate (3 × 5 mL). Ethyl acetate was then evaporated in a rotary evaporator under reduced pressure to yield 3a as a yellow crystalline solid (0.701 g, 93.4%). The compound was spectroscopically pure, and the melting point data confirmed the bulk purity.

## Results and discussion

### Characterization of CS catalyst

The CS catalyst was characterized by Fourier-transform infrared spectroscopy (FTIR) and powder X-ray diffraction (PXRD). The morphology of the CS catalyst was studied by scanning electron microscopy (SEM). The thermal stability of the CS catalyst was studied by thermogravimetric analysis (TGA) and differential scanning calorimetry (DSC). The recycled CS catalyst was also characterized and compared with fresh CS.

**Fig. 2:**
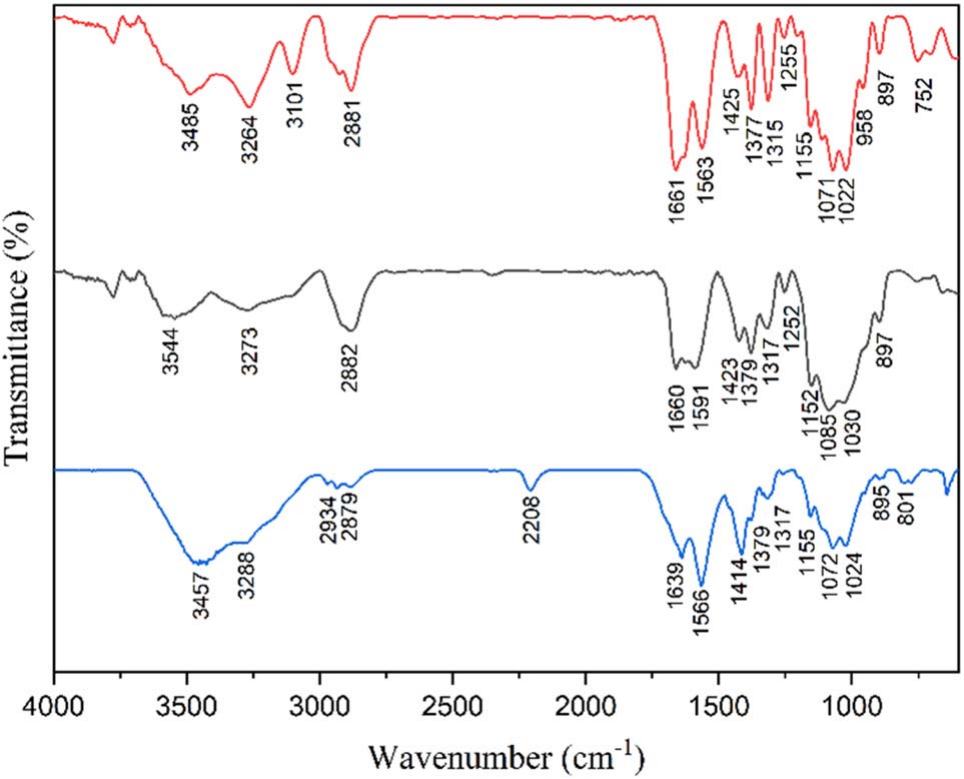
Fourier-transform infrared spectrum of chitin (red), CS (black), and recycled CS (blue) samples.

Chitin shows an O–H stretching peak at 3485 cm^−1^ and N–H peak at 3264 cm^−1^ in the FTIR spectrum (Fig. 2), which shows an acetylated structure. The peaks around 2881 cm^−1^ are responsible for the symmetric and asymmetric C–H stretching vibrations of the methylene and methyl group. The carbonyl stretching frequency of amide appears at 1661 cm^−1^. The N–H bending and C–N stretching appears around 1563 cm^−1^, whereas the 1425 cm^−1^ peak is responsible for CH_2_ bending and CH_3_ deforming. The 1155 cm^−1^ peak shows asymmetric C–O–C stretching (glycosidic bond). The FTIR spectrum of the CS catalyst showed a peak at 3273 cm^−1^, which indicates the presence of primary amine and hydroxyl groups associated with the pyranose ring. The 1660 cm^−1^ peak shows carbonyl stretching frequency of the amide group and 1591 cm^−1^ shows NH in NHCOCH_3_, whereas 1423 cm^−1^ shows CH_2_ in CH_2_OH. The CH_3_ in NHCOCH_3_ peak appears at 1379 cm^−1^, 1317 cm^−1^ indicates C–H in the pyranose ring, 1152 cm^−1^, 1085 cm^−1^ indicate C–O–C glycosidic linkage, and 897 cm^−1^ indicates pyranose ring skeletal vibration.^29,30^ The FTIR spectrum of recycled CS peaked at 2208 cm^−1^, which hints at the structural changes of recycled CS.

**Fig. 3:**
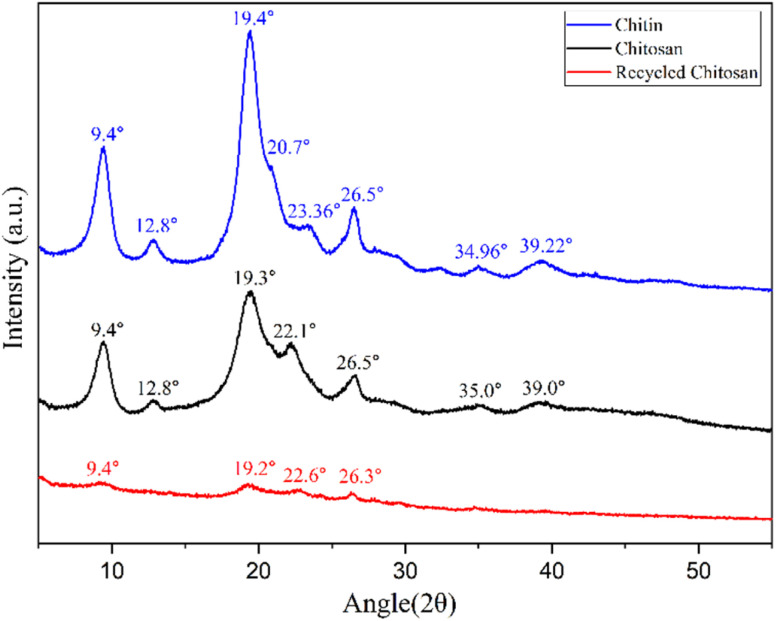
Powder X-ray diffraction analysis of chitin (blue), CS (red), and recycled CS (black).

The X-ray diffraction pattern was recorded to confirm the crystalline structure of chitin, CS, and recycled CS (Fig. 3). CS is a semi-crystalline biopolymer, which show characteristic XRD patterns depending on the degree of deacetylation.^31^ Chitin shows high crystallinity with well-defined peaks at 9.4° and 19.4°, which correspond to the intermolecular (020) and intramolecular (130) planes. This XRD pattern is typical of α-chitin, where chains are antiparallel and tightly packed. Deacetylation of chitin into CS reduces hydrogen bonding, which leads to a more amorphous structure. The broad peak at 19.3° corresponds to the plane (130) that signifies a short-range order. The JCPDS card number is 39-1894 for chitin, and the JCPDS card number for CS is 35-1974.^32^ The similarity in the peaks of chitin and CS indicates that the deacetylation process was not quantitative and it did not disrupt the polymer's crystalline domains substantially. The peak near 2*θ* ≈ 23.36° in chitin and 22.1° in CS corresponds to (130) plane shows a modest intensity. The peaks' intensity in the freshly prepared CS sample is significantly higher, which indicates a well-ordered crystalline structure. After utilizing CS as a catalyst for six cycles, low-intensity peaks were observed, showing a reduction in crystallinity. The increase in amorphous character is due to the breakdown of the crystalline domains during grinding and the introduction of lattice defects or disorders. The number of coherent scattering regions may be reduced due to the disruption in the crystalline structure. Grinding reduces the size of the particles, causing the peak broadening due to the Scherrer effect.^33^ This surface effect reduces peak intensity, sometimes creating new or less intense peaks. The crystallite size of the freshly prepared CS was calculated to be 18 nm, whereas the recycled CS crystallite size decreased to 14 nm. Weak and diffuse reflections in between the 30–40° were also evident in both Chitin and CS. Although they are not commonly associated with chitin or CS, these peaks may arise due to the presence of secondary structural effects or minor phase impurities.^34^

**Fig. 4:**
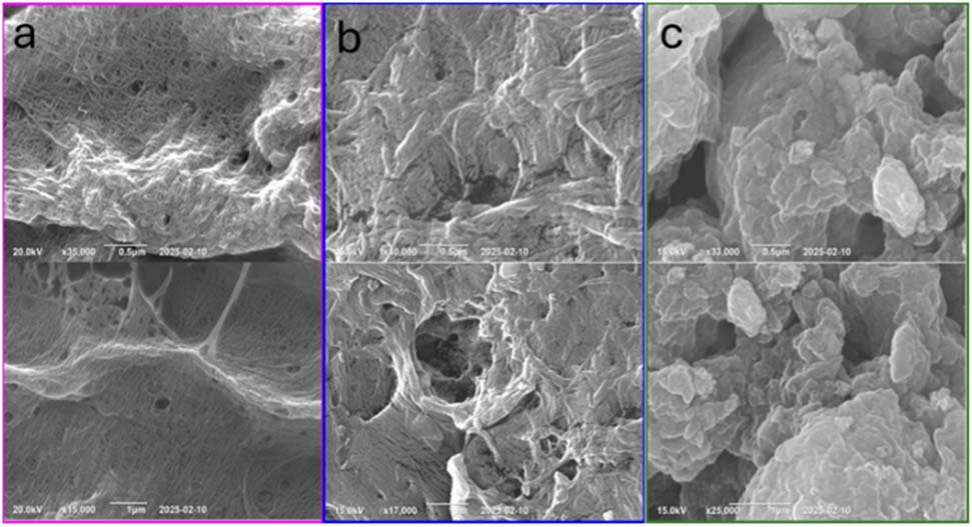
FE-SEM images of (a) chitin (pink outline), (b) fresh CS (blue outline), and (c) recycled CS (green outline) at the 0.5 μm and 1 μm scales.

FE-SEM was utilized to examine the surface morphology and structural integrity of chitin and CS samples (Fig. 4). Chitin displayed a highly fibrous, rigid, and layered structure. The fibrous arrangement of chitin is well-organized and dense, while the structural features of CS are more porous and less compact than those of chitin. The FE-SEM images of recycled CS reveal irregular and fragmented patterns, showing more cracks and rough surfaces compared to freshly prepared CS due to multiple grinding cycles. This change in morphology aligns with the reduction in crystalline structure observed in the XRD (Fig. 3) and the decrease in thermal stability of CS noted in the TGA data (Fig. 6).

**Fig. 5:**
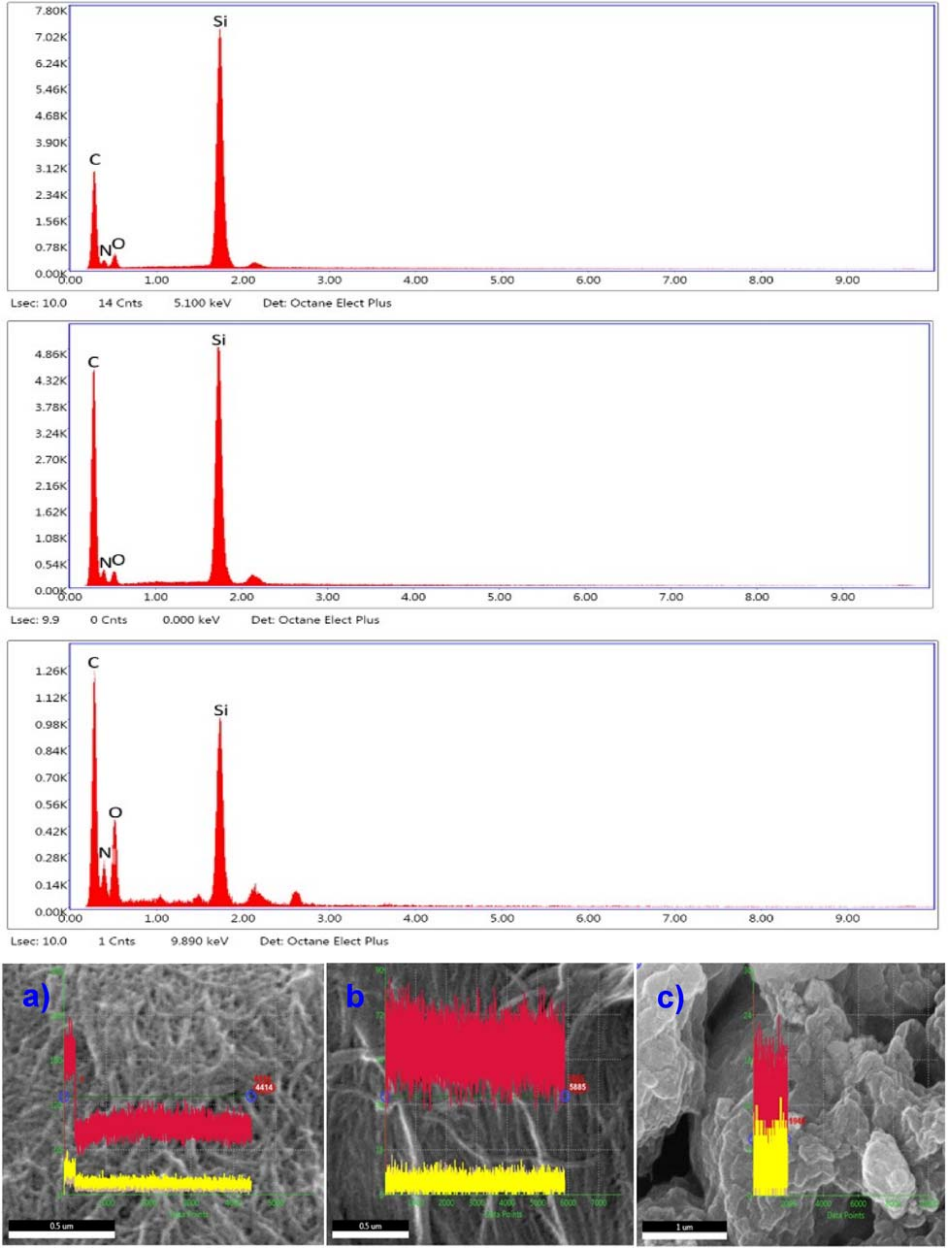
Energy dispersive spectroscopy (EDS) analysis of (a) chitin, (b) fresh CS, and (c) recycled CS.

The EDS was conducted to determine the elemental composition and chemical changes of chitin, fresh CS, and recycled CS samples (Fig. 5). The spectra revealed prominent peaks corresponding to carbon (C), nitrogen (N), and oxygen (O), which are the characteristic elements of chitin and CS. Chitin exhibited strong signals of carbon (C), which is indicated in red color, oxygen (O) in yellow color, and cream color for nitrogen (N), displayed the highest overall intensity, indicating a relatively dense organic matrix and consistent with chemical structure of chitin, which is composed of *N*-acetylglucosamine units. In the CS sample, the elemental peaks were moderately intense, reflecting partial deacetylation of chitin and a slight alteration in composition. Notably, the nitrogen peak remained prominent, which suggests the presence of amine groups after deacetylation. The recycled CS exhibited significantly lower peak intensities for all elements, which may indicate the partial degradation or structural changes during the recycling process. The increase in oxygen signals in recycled CS shows the structural changes of CS after its recycling. These findings confirm the successful transformation of chitin to CS and also provide information about the structural characteristics of recycled CS.

**Fig. 6:**
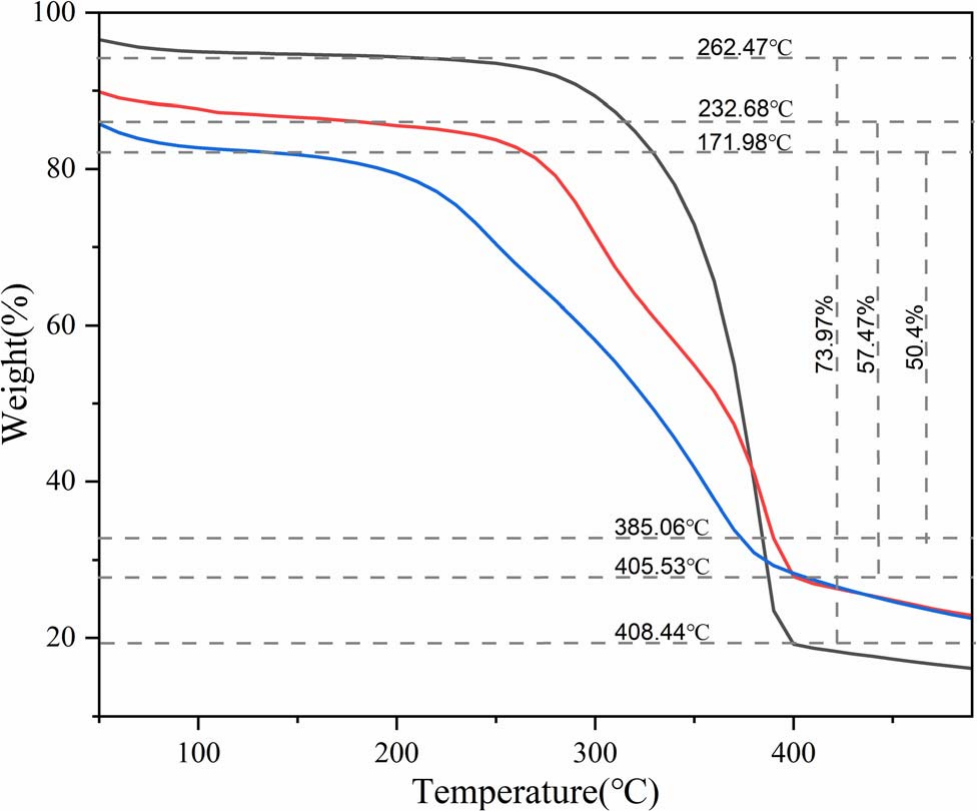
TGA curve of chitin (black), fresh CS (red), and recycled CS (blue).

TGA data shows the decomposition behaviour of chitin, CS, and recycled CS samples (Fig. 6). The effect of deacetylation and thermal stability of the recycled CS can be compared with CS and chitin. The two endothermic peaks are indicated on the thermogravimetric curves. The loss of weight in the temperature range of 50–100 °C indicates the loss of water. Weight loss occurred at a higher temperature range of 170–410 °C, hinting at the polymers' thermal stability.^35^ The initial decomposition temperatures of the chitin, CS, and recycled CS is 262.47 °C, 232.68 °C, and 171.98 °C, respectively. These peaks indicate the onset of weight loss. In contrast, chitin shows the highest thermal stability compared to CS due to its highly crystalline nature and partial deacetylation. The maximum rate of decomposition of chitin, CS, and recycled CS occurs at 408.44 °C, 405.53 °C, and 385.06 °C, respectively. The weight loss of chitin, CS, and recycled CS is 73.97%, 57.47%, and 50.4%, respectively.

**Fig. 7:**
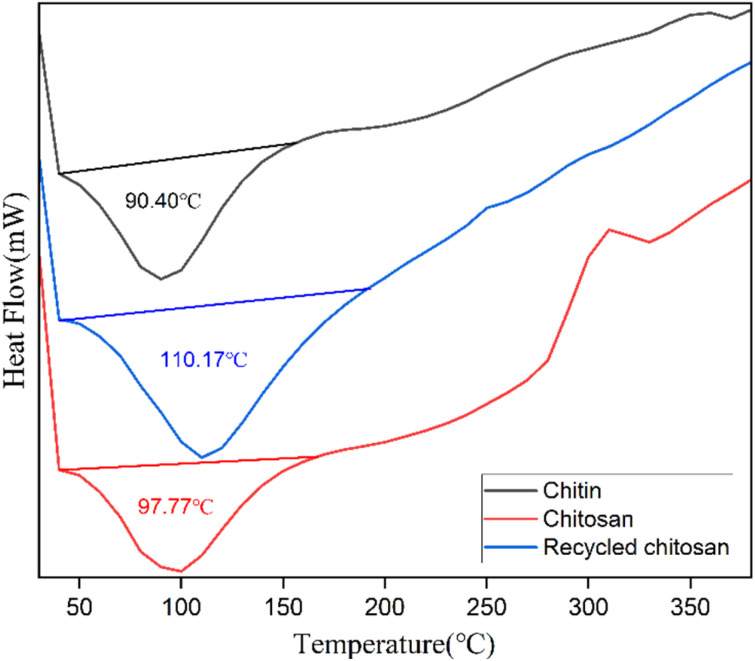
Differential scanning calorimetric analyses of CS.

DSC is used to investigate phase transitions, thermal stability, and crystallinity of chitin, CS, and recycled CS (Fig. 7). This technique is used to study the thermal behaviour of the samples and the impact of deacetylation and recycling on their structural integrity. The DSC data showed two distinct degradation stages in chitin and CS and recycled CS. The large endothermic peaks of chitin, CS, and recycled CS show peak temperatures at 90.40 °C, 97.77 °C, and 110.17 °C, respectively.

**Fig. 8:**
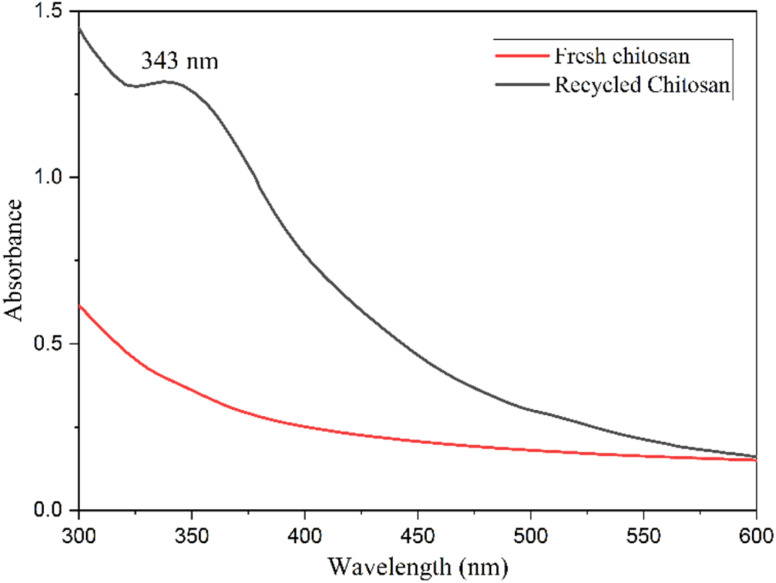
UV-Vis spectrum of fresh CS (red) and recycled CS (black).

UV-Vis spectra of the CS (fresh) and CS (6th recycle) samples dissolved in 2% acetic acid aqueous solution were recorded. While the fresh CS catalyst appeared colorless in the solution, the recycled CS gave a yellow color solution (Fig. S33, ESI†). Fig. 8 shows noticeable absorption around 325–375 nm, possibly due to the colored impurities absorbed on the CS catalyst.

### Synthesis of Knoevenagel condensation products using CS catalyst

**Fig. 9:**
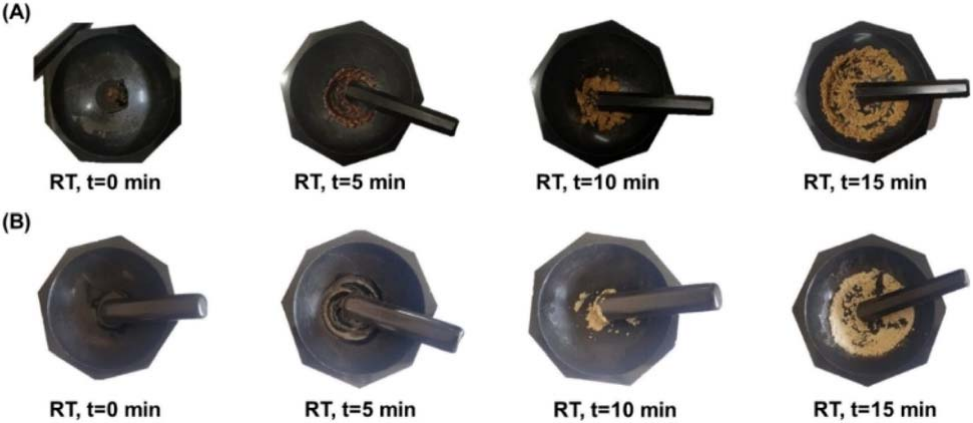
Evolution of the Knoevenagel condensation reaction between (A) furfural 1a and malononitrile 2, and (B) benzaldehyde 1 h and malononitrile 2 by mechanochemistry using CS (10 wt%) as a catalyst.

Chitin was then deacetylated by refluxing in 45% aqueous NaOH for 1–3 days. The CS produced was washed copiously with deionized water and dried in a hot-air oven (80 °C) for 12 h before using it as a catalyst. Initially, the Knoevenagel condensation reaction between furfural (1a) and malononitrile (2) was attempted using 20 wt% (compared to furfural) of the CS catalyst. Equimolar amounts of 1a and 2 were taken in a granite mortar, and CS powder was added (Fig. 9A). The mixture was continuously ground using a granite pestle. The reaction was monitored by thin-layer chromatography (TLC) for the disappearance of 1a. The liquid became a paste within a few minutes and then solidified within 3 minutes. The reaction duration was 5–7 minutes, depending on the scale of the reaction. In a control reaction, equimolar amounts of 1a and 2 were ground for 30 min, but the Knoevenagel condensation product 3a formation was not discernible. When the CS catalyst loading was decreased to 10 wt%, the reaction duration was increased from 5 min to 9 min, and no significant change in the yield of 3a was observed. When the amount of 1a was increased to 1 g scale using 10 wt% CS catalyst, the reaction was completed in 9 min and afforded a 93.4% yield of 3a. The solid product was then transferred to a beaker and dissolved in ethyl acetate. The CS catalyst was filtered, washed with ethyl acetate, and dried in a hot-air oven (80 °C, 6 h) before subjecting it to the next catalytic cycle. The recycled CS catalyst was slightly colored, but the activity remained the same in the subsequent cycles (up to the fifth cycle). When benzaldehyde (1h) was used as the substrate, the reaction was completed in 15 min at room temperature using only 10 wt% of the CS catalyst (Fig. 9B). The CS catalyst prepared by deacetylation reaction for 72 h showed better catalytic activity than that prepared over 24 h. The observation can be explained by a higher degree of acetylation after 72 h, making it a better base catalyst. No reaction between 1a and 2 was observed when chitin was used as the catalyst.

Table 1 lists the Knoevenagel condensation products reported in this work. Compounds 3a–3g (entries 1–7) are produced by reacting carbohydrate-derived 5-substituted-2-furaldehydes 1a–1g with malononitrile 2.

**Table 1:** Molecular structure, code, duration, and yield of the Knoevenagel products reported in this work^a^

Entry	Product	Duration & yield (%)	Entry	Product	Duration & yield (%)
1	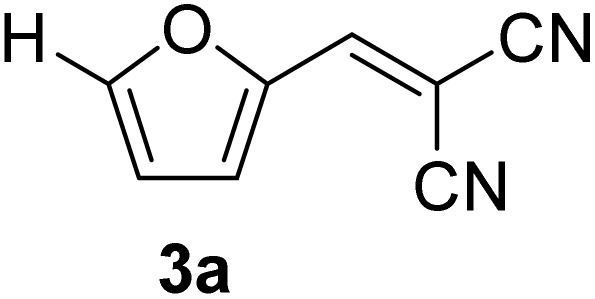	9 min (93.4%)	6	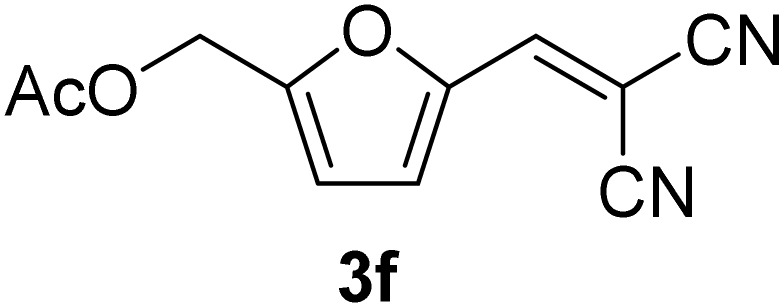	12 min (93.6%)
2	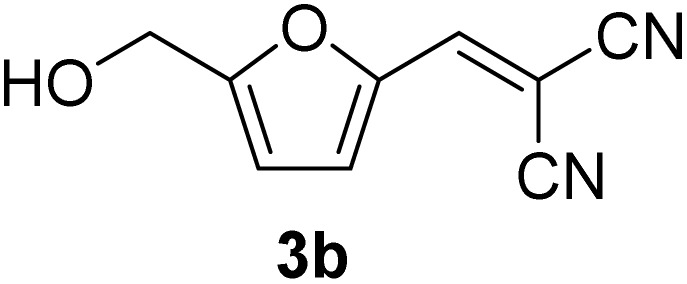	12 min (92.6%)	7	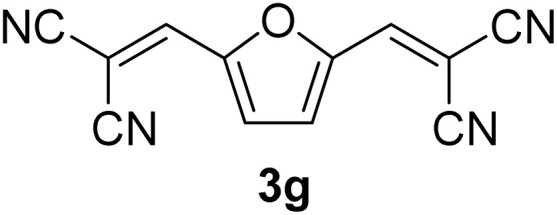	15 min (92.3%)
3	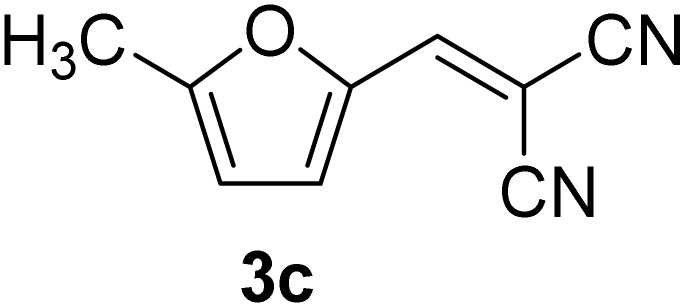	9 min (94.9%)	8	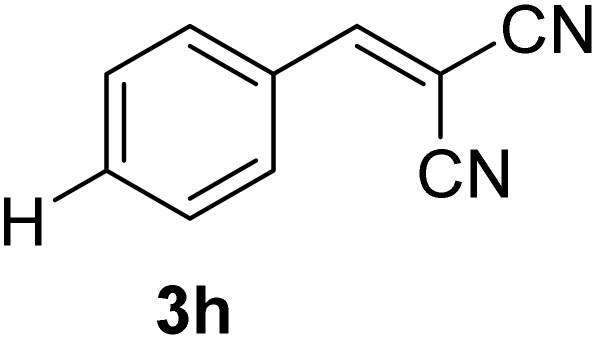	15 min (94.4%)
4	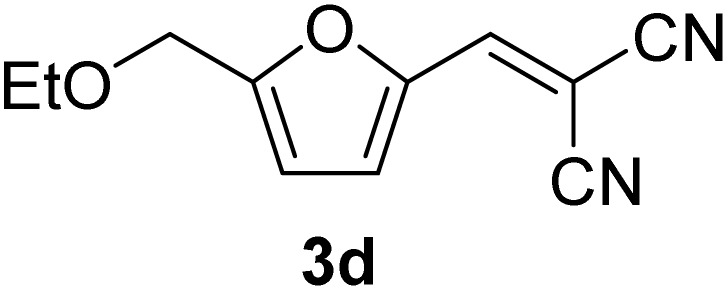	11 min (93.5%)	9	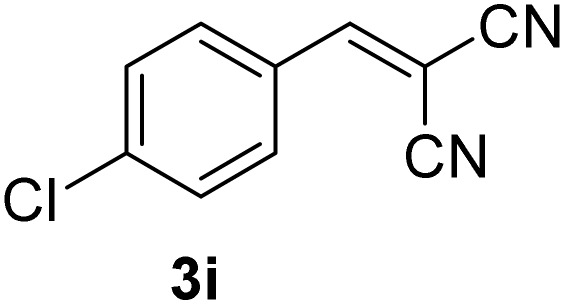	20 min (93.1%)
5	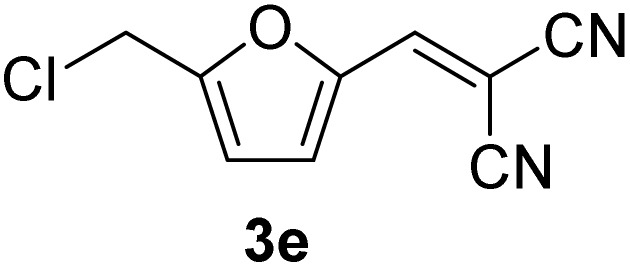	9 min (94.4%)	10	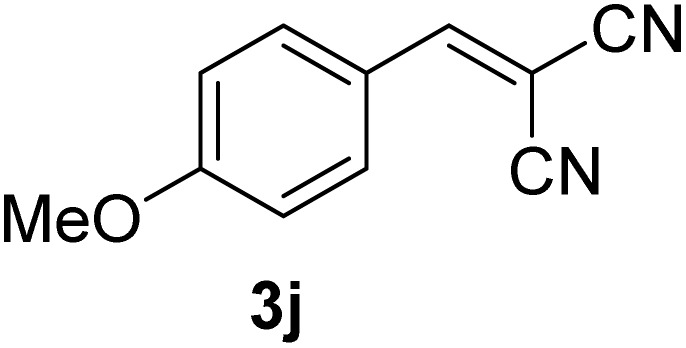	25 min (90.3%)

a Reaction conditions: aldehyde (0.500 g), malononitrile (1 eq.), CS (0.050 g, 10 wt%), grounded in mortar and pestle.

**Fig. 10:**
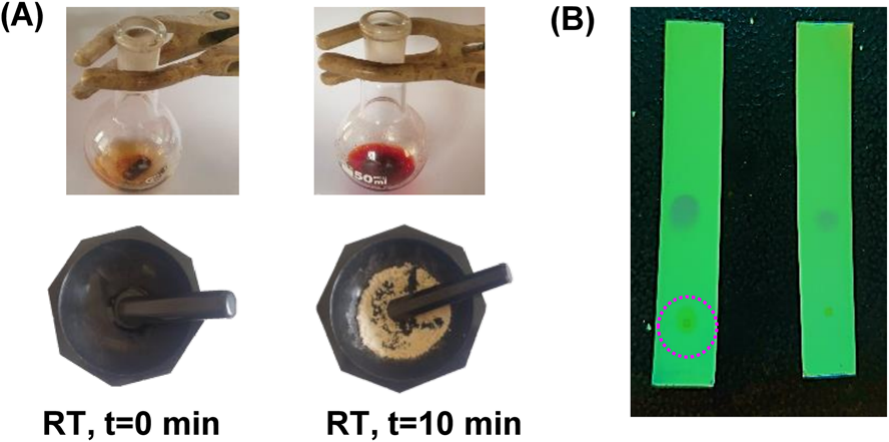
Photographic images of (A) Knoevenagel condensation reaction between furfural 1a and malononitrile 2 in ethanol (top) and under mechanochemistry (bottom), and (B) TLC of the reaction mixture in ethanol (left) and mechanochemistry (right).

The transformations were completed in 9–15 minutes at RT using equivalent amounts of 1a–1g with 2 using CS as the organocatalyst under mechanochemical conditions. Interestingly, when CMF, 1e was used as the substrate (entry 5), the corresponding Knoevenagel condensation product was formed within 9 min at RT, affording a 94.4% isolated yield of 3e. The chloromethyl group in CMF 1e remained intact. Therefore, 3e can be reacted with nucleophiles like alcohols and amines to make novel renewable chemicals. When DFF, 1g was used as the substrate and reacted with two equivalents of 2, 3g was isolated in a 92.3% yield after a 15 min reaction at RT (entry 7). Benzaldehyde 1h afforded a 94.4% isolated yield of the corresponding Knoevenagel condensation product 3h (entry 8) after 15 min reaction at RT. When 4-chlorobenzaldehyde (1i) and 4-methoxybenzaldehyde (1j) were used as substrates, the reaction took slightly longer to complete but afforded excellent yields (>90%) of the corresponding Knoevenagel condensation products 3i and 3j (entries 9&10). The colored impurity formed in solvent-assisted synthesis versus solvent-free mechanochemistry were evident visibly and on the TLC plate in the Knoevenagel condensation reaction between 1a and 2 using CS (10 wt%) as the organocatalyst (Fig. 10).

**Scheme 1:**
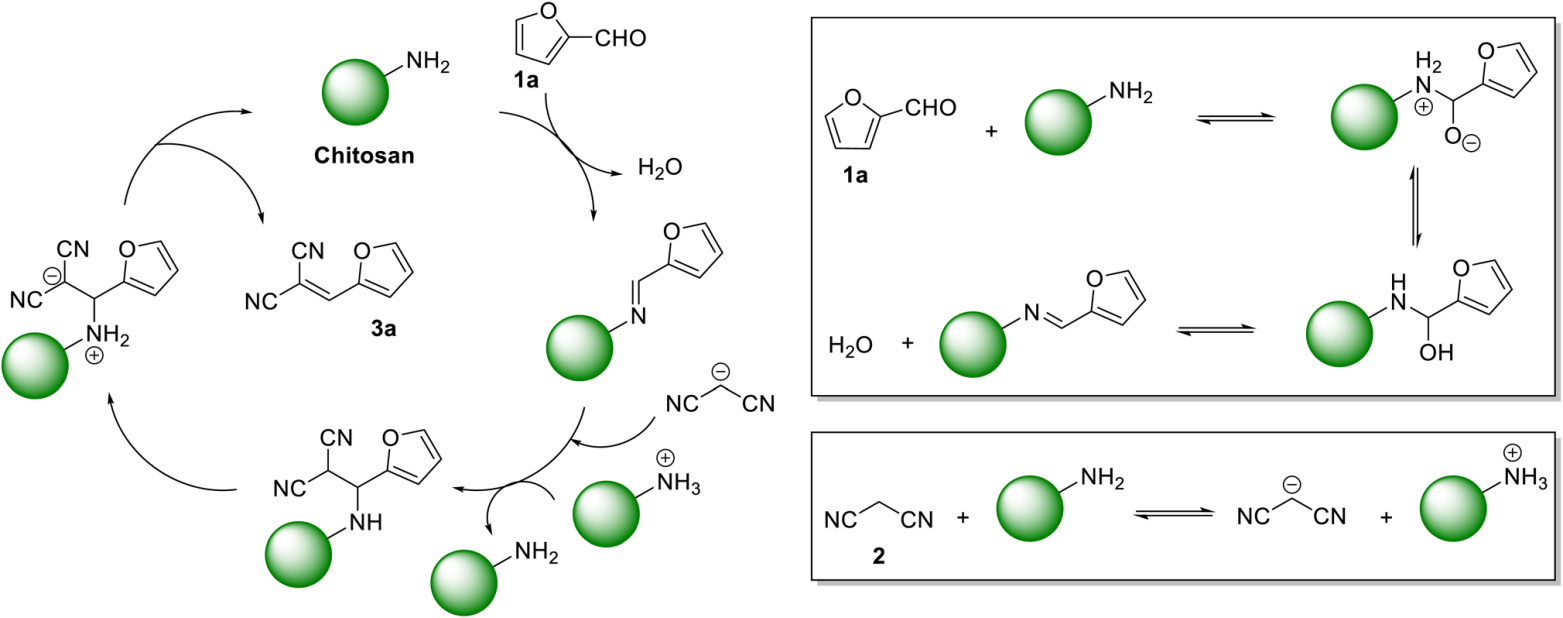
The proposed mechanism of forming the Knoevenagel condensation product 3a using CS as the organocatalyst.

Even though the mechanism of Knoevenagel condensation reaction using heterogeneous amine catalyst is known in the literature, the following mechanism is proposed for the synthesis of 3a using CS as the heterogeneous organocatalyst. In the initial step, CS is condensed with 1a to form an imine intermediate (Scheme 1). The malononitrile 2 is deprotonated by the CS catalyst. The deprotonated 2 then reacts with the imine to form 3a, releasing CS for the next catalytic cycle.

### Recyclability of the CS catalyst

**Fig. 11:**
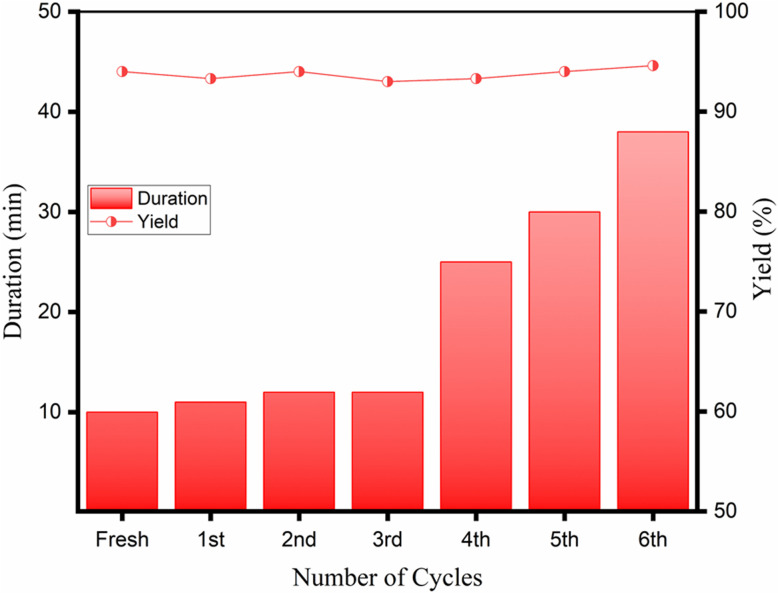
Recyclability of the CS catalyst for the mechanochemical synthesis of 3a at room temperature.

The recyclability of the CS catalyst was studied for six consecutive cycles in preparing 3a. The reaction time remained relatively stable until the third cycle (Fig. 11), indicating the catalytic efficiency was maintained. However, the reaction duration began to increase after the third cycle, and effectiveness was reduced, likely due to the structural changes of the CS. The deposition of organic impurities on the surface of the CS catalyst can justify such an observation. Regardless, the yield of 3a remained constant throughout all cycles, showing that the catalytic property of the CS remained unaltered. With each recycling, the CS catalyst became increasingly colored, which may be attributed to the absorption of the side products and colored impurities. The texture and catalytic activity of CS were intact, which confirmed that the polymeric material did not undergo degradation to any noticeable extent. The color impurity present in the catalyst was attempted to be removed after the 4th cycle. The recovered CS catalyst was refluxed in ethanol for 1 h, filtered under vacuum, washed with fresh ethanol, and finally dried in a hot-air oven. A partial recovery of the catalytic reactivity was achieved for the ethanol-washed CS catalyst, and the Knoevenagel condensation reaction was completed in noticeably less time compared to the recycled CS catalyst without ethanol wash. This observation, supported by the UV-Vis spectra of fresh and recycled CS catalysts, hints at the possibility of deposition of organic impurities on the catalyst surface.

## Conclusions

In conclusion, CS was prepared by deacetylating crustacean waste-derived chitin in an aqueous sodium hydroxide solution. CS was then used as a non-toxic, biodegradable, heterogeneous, and recyclable base catalyst for the Knoevenagel condensation reaction between carbohydrate-derived 5-substituted-2-furaldehydes and malononitrile under solvent-free mechanochemical process. The process was extended to other aromatic aldehydes to demonstrate the high substrate scope of the process. The Knoevenagel condensation products were obtained in excellent isolated yields (>85%), and the CS catalyst was recycled for six cycles without a catastrophic drop in the yield of the condensation product. The mechanochemical process had faster kinetics than solution phase synthesis, producing fewer colored impurities. Future work will explore using CS as a biorenewable catalyst for other organic transformations.

## Date availability

The data supporting this article have been included as part of the ESI.†

## Author contributions

Rachitha S N characterized the CS catalyst, optimized the reaction conditions, and analyzed the products. Abhishek Kumar Yadav synthesized the CS catalyst and edited the manuscript. Mangalapalli Kamali assisted in the FESEM, TGA, and DSC analyses and edited the manuscript. Putla Sudarsanam and Saikat Dutta conceptualized the work, supervised the progress, and wrote the original manuscript.

## Conflicts of interest

There are no conflicts to declare.

## Abbreviations

AcMF: 5-(Acetoxymethyl)furfural
CS: Chitosan
CMF: 5-(Chloromethyl)furfural
DSC: Differential scanning calorimetry
DFF: 2,5-Diformylfuran
EDX: Energy dispersive X-ray analysis
EMF: 5-(Ethoxymethyl)furfural
HMF: 5-(Hydroxymethyl)furfural
MF: 5-Methylfurfural
NMR: Nuclear magnetic resonance
PXRD: Powder X-ray diffraction
FE-SEM: Field-emission scanning electron microscopy
TGA: Thermogravimetric analysis
TLC: Thin-layer chromatography
FTIR: Fourier transform infrared

## Supplementary Material

RA-015-D5RA02836A-s001
