# Supplementary material for: Mechanochemical synthesis of Knoevenagel condensation products from biorenewable furaldehydes using crustacean waste-derived chitosan as a sustainable organocatalyst
Source: RSC Adv. 2025 Jun 10;15(25):19687–95. doi: 10.1039/d5ra02836a (PMC12150282; doi:10.1039/d5ra02836a)
Supplement: RA-015-D5RA02836A-s001 [file RA-015-D5RA02836A-s001.pdf]

Supplementary Information (SI) for RSC Advances.
This journal is © The Royal Society of Chemistry 2025

Supplementary information

Mechanochemical synthesis of Knoevenagel condensation products from biorenewable furaldehydes using crustacean waste-derived chitosan as a sustainable organocatalyst

Rachitha S N,[1] Abhishek Kumar Yadav,[1] Mangalapalli Kamali,[2] Putla Sudarsanam,[2] and Saikat Dutta[1*]

[1] Department of Chemistry, National Institute of Technology Karnataka, Surathkal, Mangalore–575025, Karnataka, India.

[2] Department of Chemistry, Indian Institute of Technology Hyderabad, Kandi–502284, Telangana, India.

[*] Corresponding author. E-mail: sdutta@nitk.edu.in

Number of Figures: 36

Number of Pages: 21

The spectroscopic, compositional, and melting point of the synthesized compounds

2-(Furan-2-ylmethylene)malononitrile(**3a**): Yellow solid (93.4%), Melting point: 70 °C, ^{1}H NMR ($CDCl_3$, 400 MHz): δ 7.80 (d, 1H, J = 1.6 Hz), 7.51 (s, 1H), 7.36 (d, 1H, J = 3.6 Hz), 6.71 (dd, 1H, J = 1.6 Hz, J = 1.6 Hz); $^{13}C\{^{1}H\}$ NMR ($CDCl_3$, 100 MHz): δ 148.66, 147.07, 142.14, 122.71, 113.49, 112.87, 111.66; FTIR (ATR, cm^{-1}): 2925, 2224, 1602.[1]

2-((5-(Hydroxymethyl)furan-2-yl)methylene)malononitrile(**3b**): Yellow oil (92.6%), ^{1}H NMR ($CDCl_3$, 400 MHz): δ 7.45 (s, 1H), 7.24 (t, 1H, J = 3.6 Hz), 6.58 (d, 1H, J = 4.0 Hz), 4.65 (s, 2H), 3.50 (s, 1H); $^{13}C\{^{1}H\}$ NMR ($CDCl_3$, 100 MHz): δ 162.96, 147.48, 142.99, 125.57, 113.95, 112.97, 111.85, 75.72, 57.11; FTIR (ATR, cm^{-1}): 3455, 2957, 2226, 1606.[1]

2-((5-Methylfuran-2-yl)methylene)malononitrile(**3c**): Yellow solid (94.9%), Melting point: 94 °C, ^{1}H NMR ($CDCl_3$, 400 MHz): δ 7.46 (s, 1H), 7.34 (d, 1H, J = 4.4 Hz), 6.45 (d, 1H, J = 3.6 Hz), 2.55 (s, 3H); $^{13}C\{^{1}H\}$ NMR ($CDCl_3$, 100 MHz): δ 161.99, 146.87, 142.15, 126.01, 114.35, 113.03, 111.79, 74.12, 14.25; FTIR (ATR, cm^{-1}): 2923, 2209, 1603.[1]

2-((5-(Ethoxymethyl)furan-2-yl)methylene)malononitrile(**3d**): Yellow oil (93.5%), ^{1}H NMR ($CDCl_3$, 400 MHz): δ 7.51 (s, 1H), 7.34 (d, 1H, J = 2.8 Hz), 6.64 (d, 1H, J = 3.6 Hz), 4.56 (s, 1H), 3.63 (q, 2H, J = 6.8 Hz), 1.24 (t, 3H, J = 6.8 Hz); $^{13}C\{^{1}H\}$ NMR ($CDCl_3$, 100 MHz): δ 160.69, 147.46, 142.7, 124.58, 113.86, 112.63, 112.59, 76.23, 66.69, 64.38, 14.86; FTIR (ATR, cm^{-1}): 2971, 2225, 1608.[1]

2-((5-(Chloromethyl)furan-2-yl)methylene)malononitrile(**3e**): Light yellow solid (94.4%), Melting point: 73 °C, ^{1}H NMR ($CDCl_3$, 400 MHz): δ 7.48 (s, 1H), 7.33 (d, 1H, J = 3.6 Hz), 6.68 (d, 1H, J = 3.6 Hz), 4.62 (s, 3H); $^{13}C\{^{1}H\}$ NMR ($CDCl_3$, 100 MHz): δ 157.64, 148.16, 142.75, 124.007, 113.668, 113.63, 112.45, 78.35, 36.29; FTIR (ATR, cm^{-1}): 2920, 2227, 1607, 722.[1]

(5-(2,2-Dicyanovinyl)furan-2-yl)methyl acetate(**3f**): Yellow solid (93.6%), Melting point: 76 °C, ^{1}H NMR ($CDCl_3$, 400 MHz): δ 7.48 (s, 1H), 7.32 (d, 1H, J = 3.2 Hz), 6.66 (d, 1H, J = 3.6 Hz), 5.13 (s, 1H), 2.1 (s, 1H); $^{13}C\{^{1}H\}$ NMR ($CDCl_3$, 100 MHz): δ 170.2, 157.2, 147.9, 142.82, 124.12, 113.9, 113.73, 112.46, 77.71, 57.53, 20.55. FTIR (ATR, cm^{-1}): 2924, 2227, 1742, 1608.[1]

2,2'-(Furan-2,5-diylbis(methaneylylidene))dimalononitrile(**3g**): Dark red solid (92.3%), Melting point: 207 °C, ^{1}H NMR (DMSO-d_6, 400 MHz): δ 7.47 (s, 1H), 6.67 (s, 1H); ^{13}C{^{1}H} NMR (DMSO-d_6, 100 MHz): δ 152.13, 144.25, 125.45, 114.29, 112.88, 81.91; FTIR (ATR, cm^{-1}): 2922, 2223, 1590.[1]

2-Benzylidenemalononitrile(**3h**): White solid (94.4%), Melting point: 87°C, ^{1}H NMR ($CDCl_3$, 400 MHz): δ 7.91 (d, 2H, *J* = 7.6 Hz), 7.78 (s, 1H), 7.63 (t, 1H, *J* = 7.6 Hz), 7.54 (t, 2H, *J* = 7.2 Hz); ^{13}C{^{1}H} NMR ($CDCl_3$, 100 MHz): δ 160.10, 134.82, 131.15, 130.93, 129.84, 113.89, 83.16; FTIR (ATR, cm^{-1}): 2928, 2224, 1589.

2-(4-Chlorobenzylidene)malononitrile(**3i**): Light yellow solid (93.1%), Melting point: 168°C, ^{1}H NMR ($CDCl_3$, 400 MHz): δ 7.85 (d, 2H, *J* = 8.8 Hz), 7.72 (s, 1H), 7.52 (d, 2H, *J* = 8.8 Hz); ^{13}C{^{1}H} NMR ($CDCl_3$, 100 MHz): δ 158.45, 141.39, 132.04, 130.30, 129.49, 113.63, 83.63; FTIR (ATR, cm^{-1}): 2920, 2225, 1584.

2-(4-Methoxybenzylidene)malononitrile(**3j**): Light yellow solid (90.3%), Melting point: 120°C, ^{1}H NMR ($CDCl_3$, 400 MHz): δ 7.91 (d, 2H, *J* = 8.8 Hz), 7.65 (s, 1H), 7.01 (d, 2H, *J* = 8.8 Hz), 3.9 (s, 3H); ^{13}C{^{1}H} NMR ($CDCl_3$, 100 MHz): δ 165.03, 159.05, 133.65, 124.24, 115.34, 114.62, 78.83, 56.01; FTIR (ATR, cm^{-1}): 2919, 2221, 1568.

The FTIR, ^{1}H NMR, ^{13}C{^{1}H} NMR, and melting point data of synthesized compounds **3a**-**3g** are matching with the literature.[1]

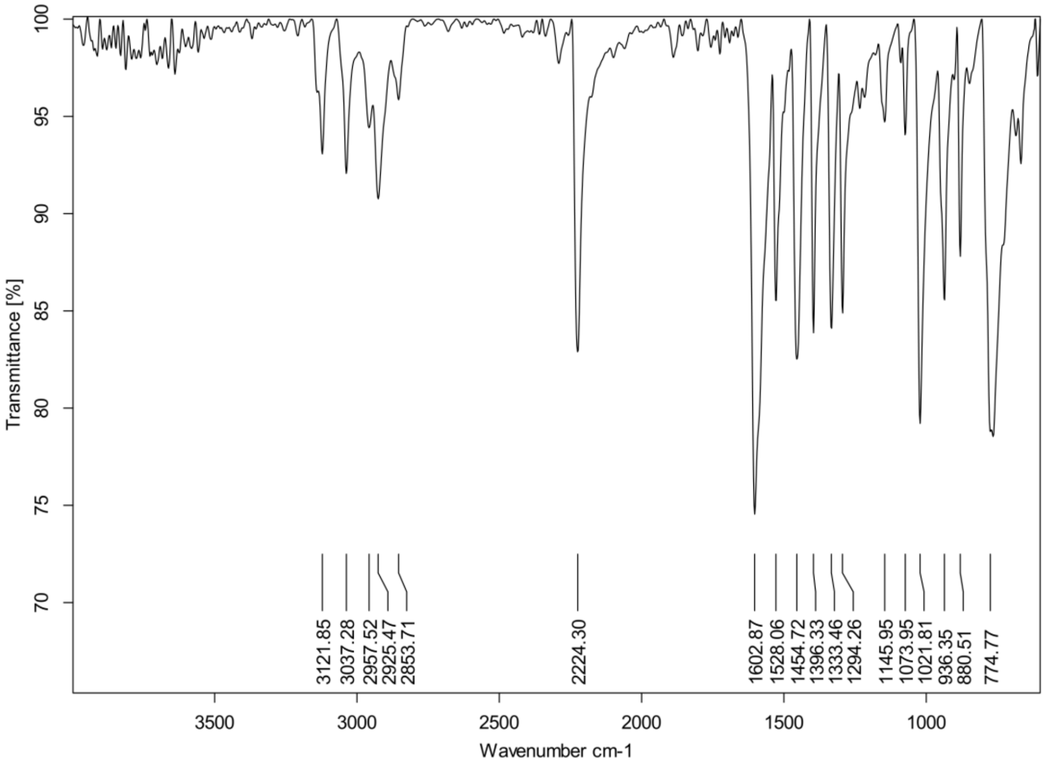

Figure S1. The FTIR spectrum of **3a.**

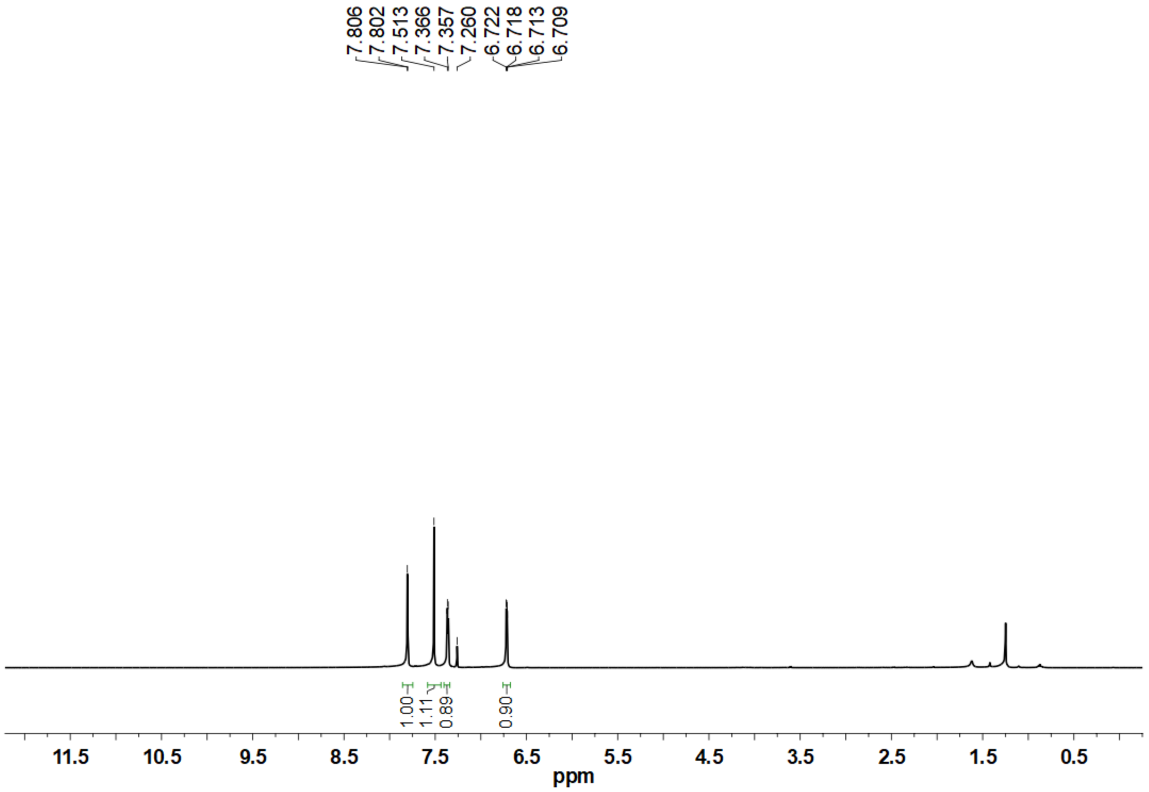

Figure S2. The ^{1}H-NMR spectrum of **3a**.

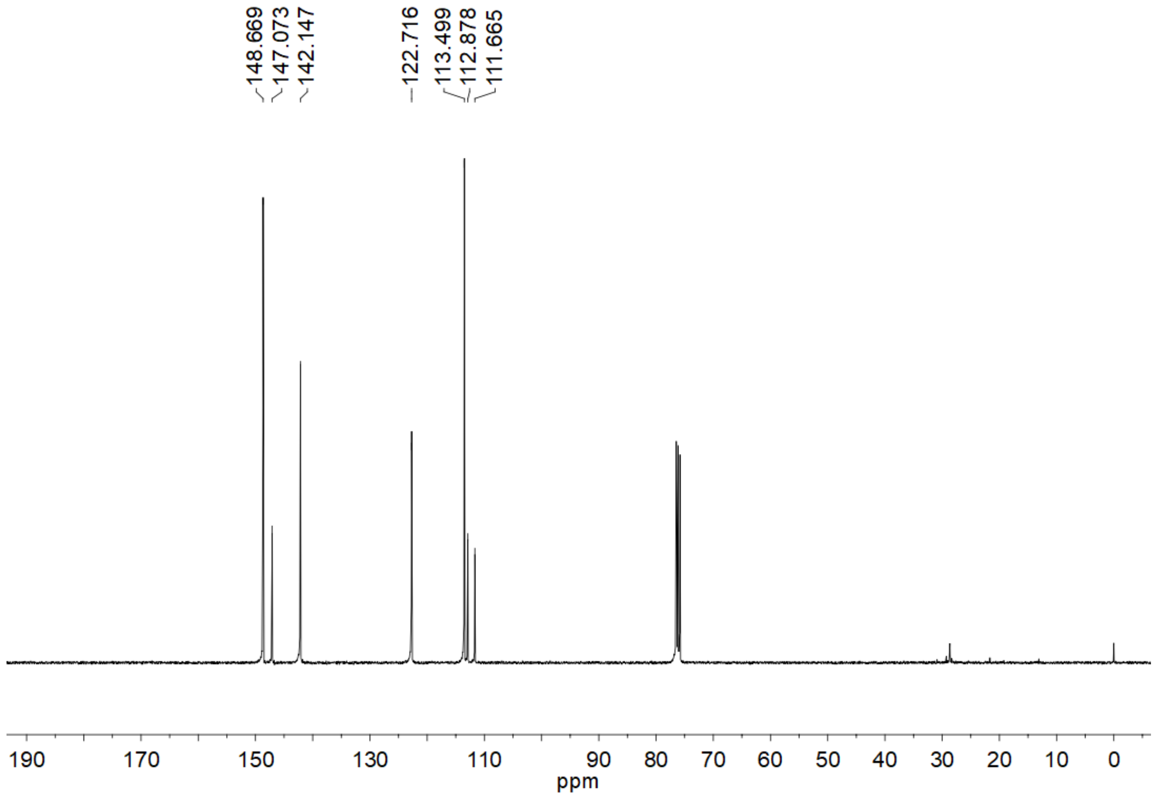

Figure S3. The ^{13}C-NMR spectrum of **3a**.

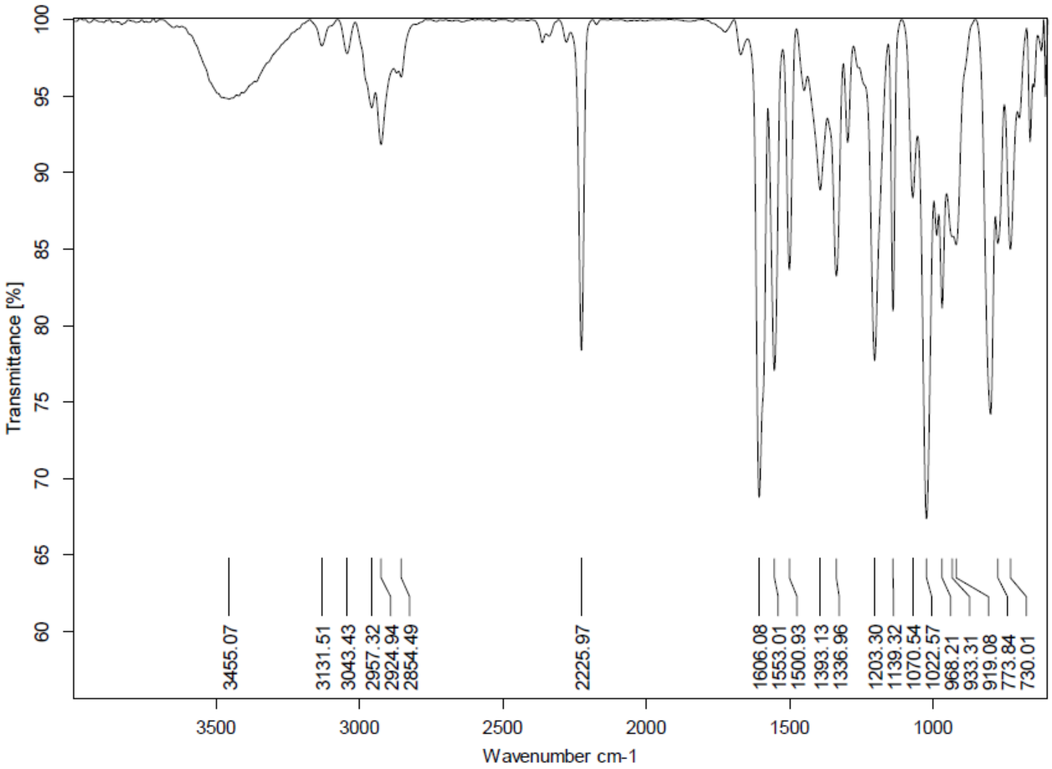

Figure S4. The FTIR spectrum of **3b.**

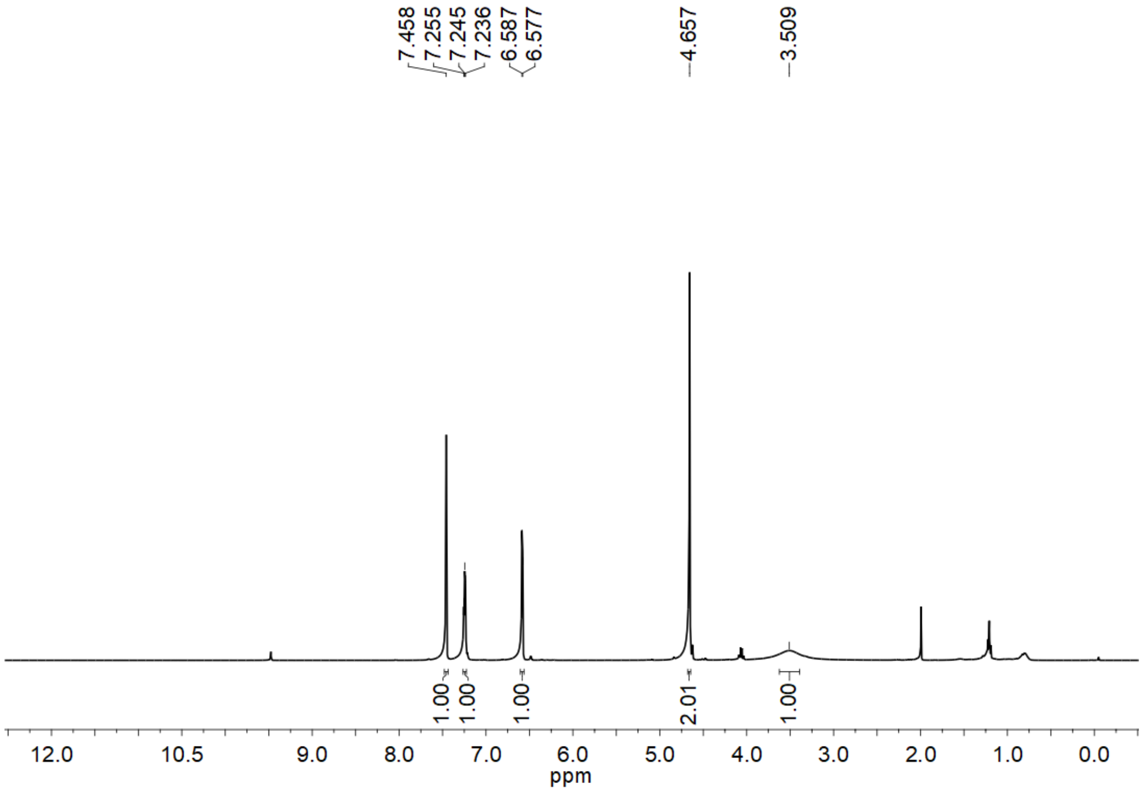

Figure S5. The ^{1}H-NMR spectrum of **3b**.

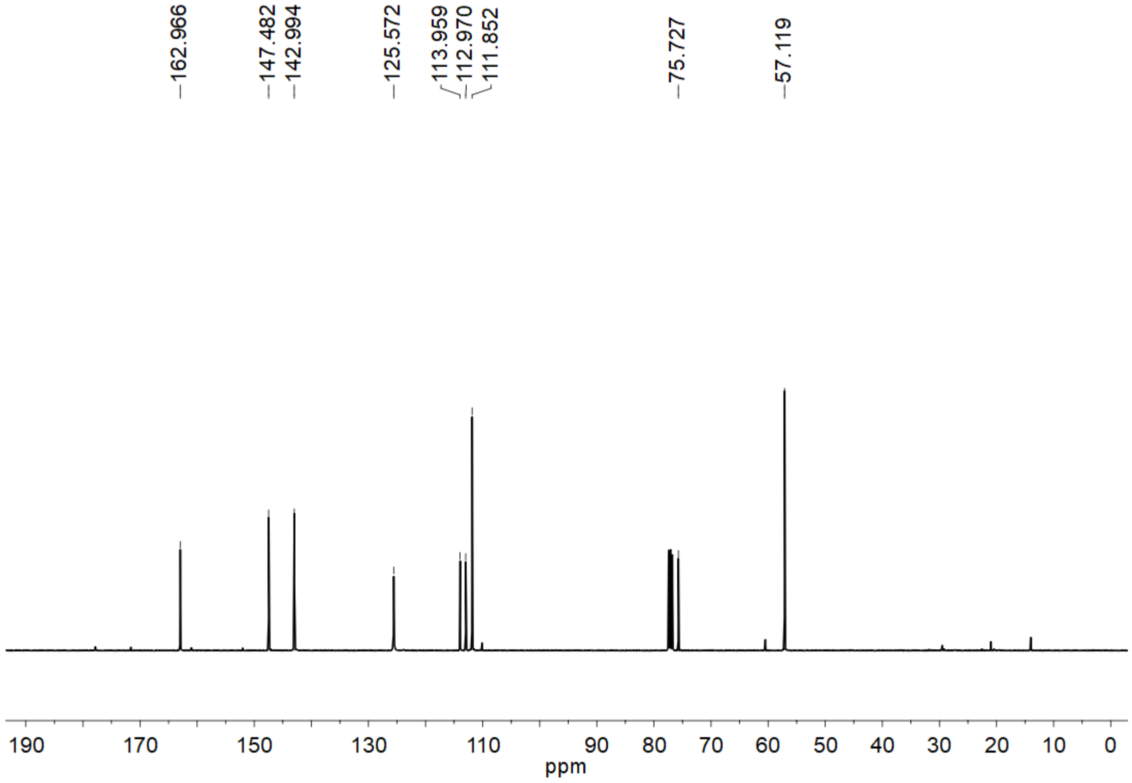

Figure S6. The ^{13}C-NMR spectrum of **3b**.

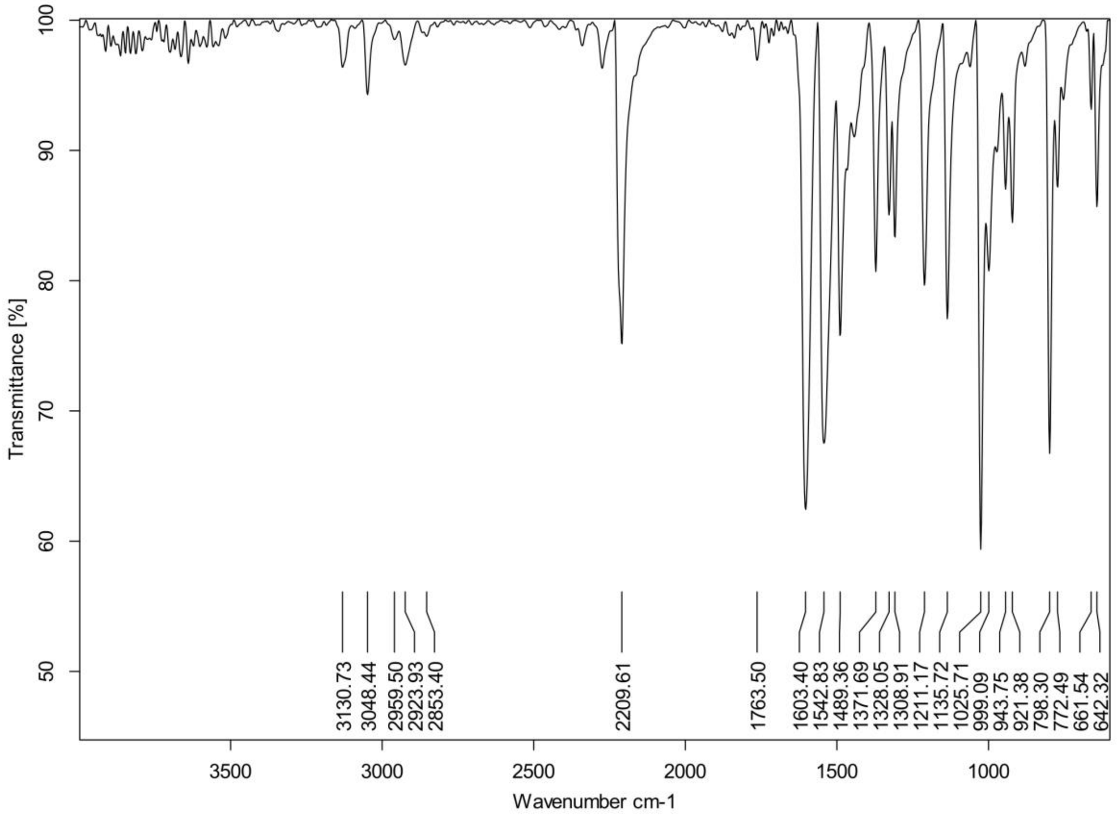

Figure S7. The FTIR spectrum of **3c.**

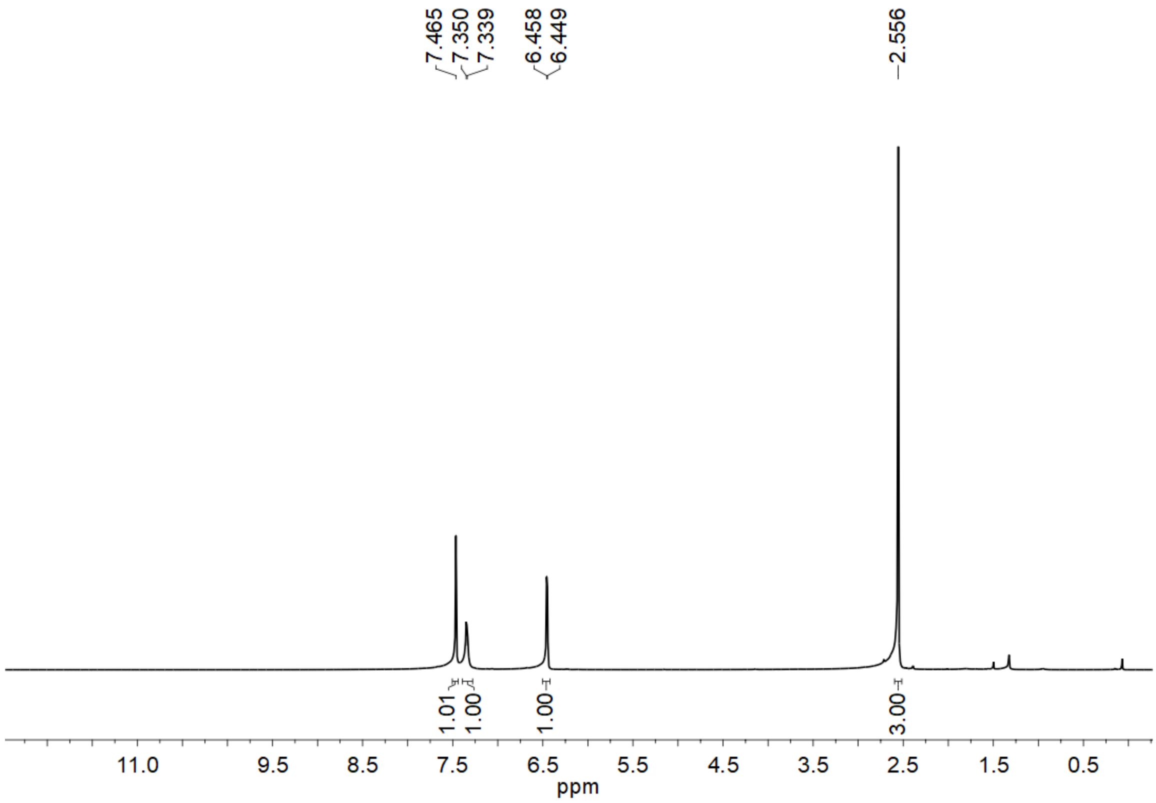

Figure S8. The ^{1}H-NMR spectrum of **3c**.

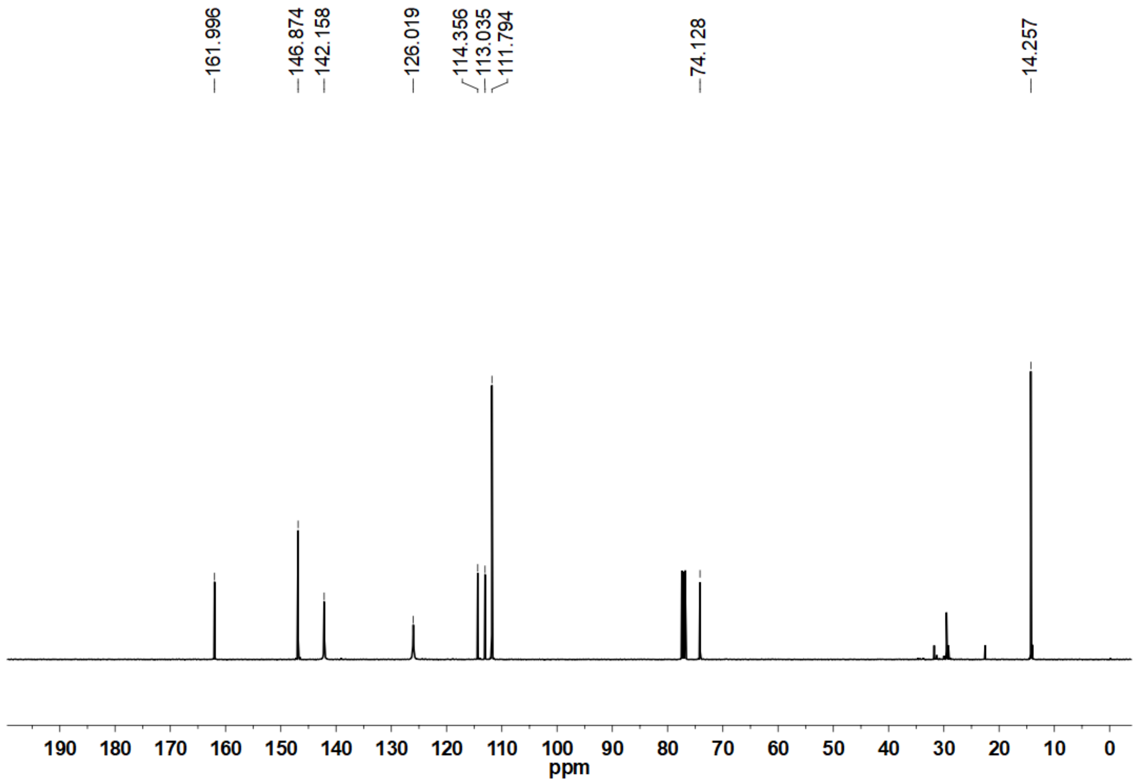

Figure S9. The ^{13}C-NMR spectrum of **3c**.

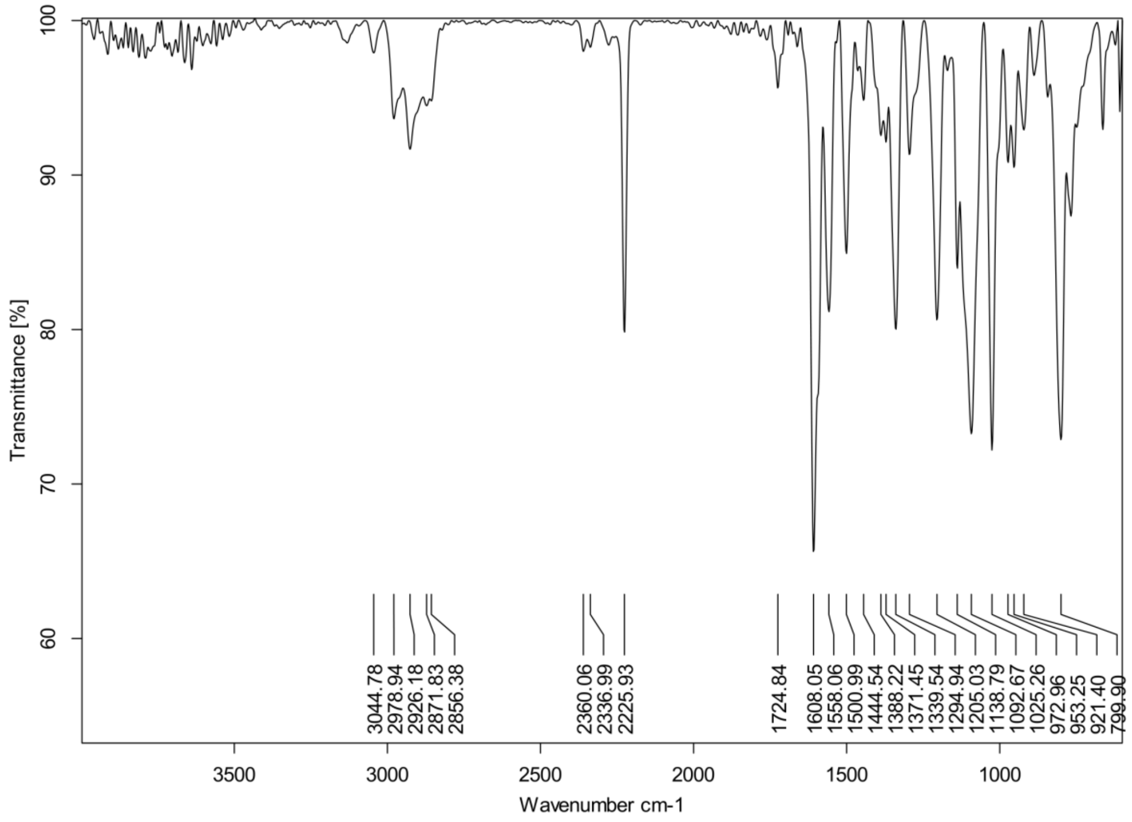

Figure S10. The FTIR spectrum of **3d.**

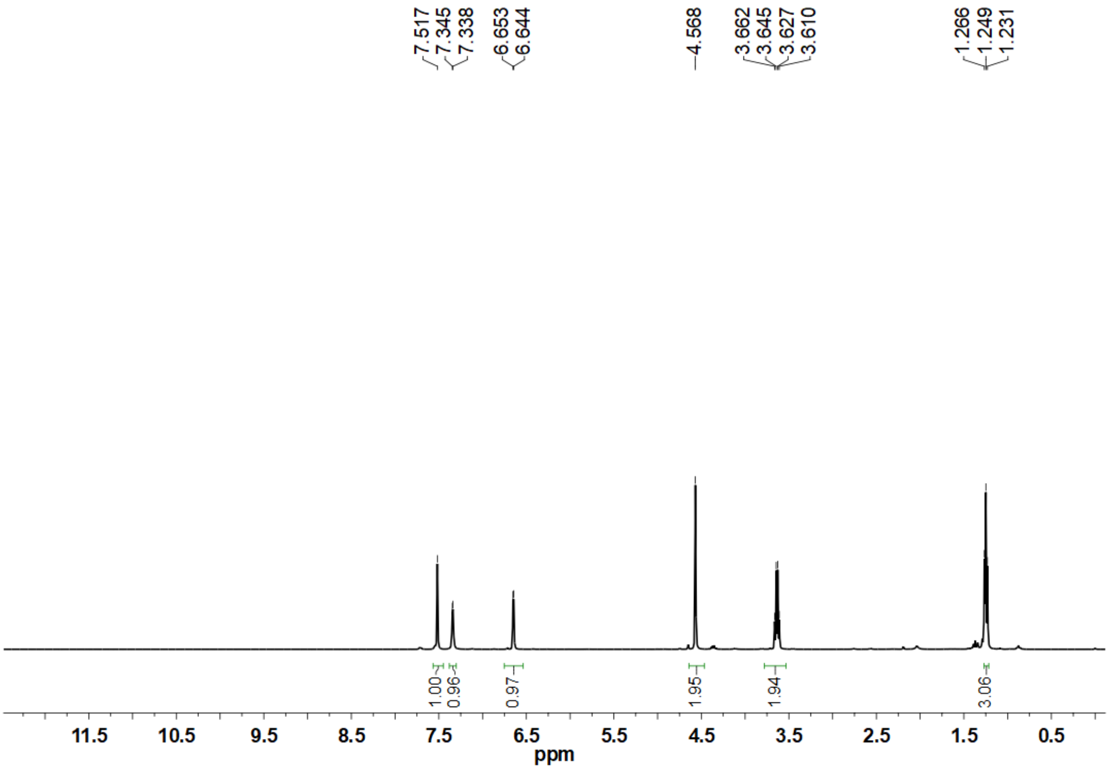

Figure S11. The ^{1}H-NMR spectrum of **3d**.

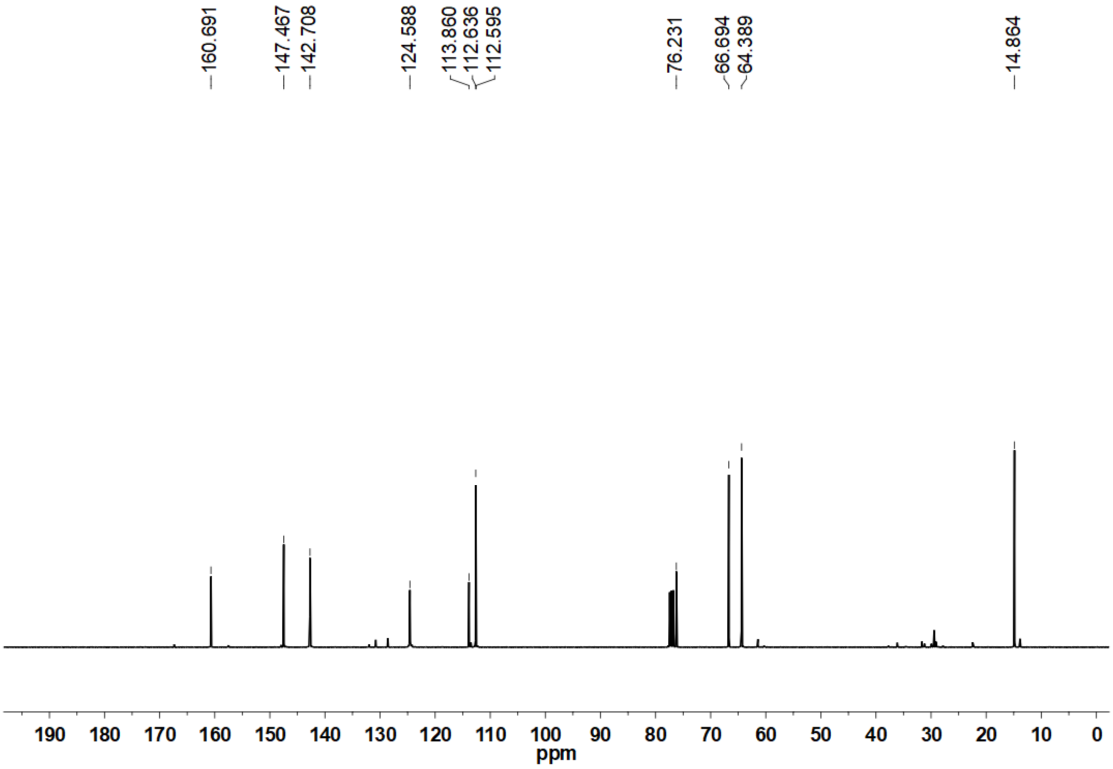

Figure S12. The ^{13}C-NMR spectrum of **3d**.

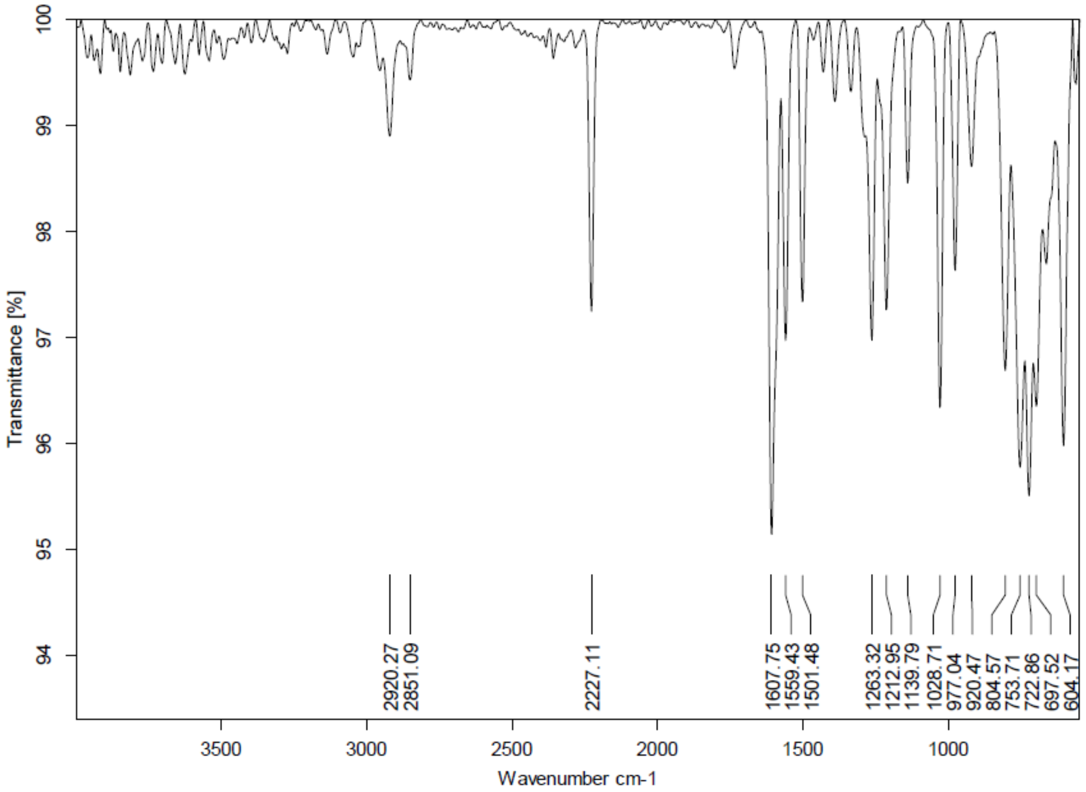

Figure S13. The FTIR spectrum of **3e.**

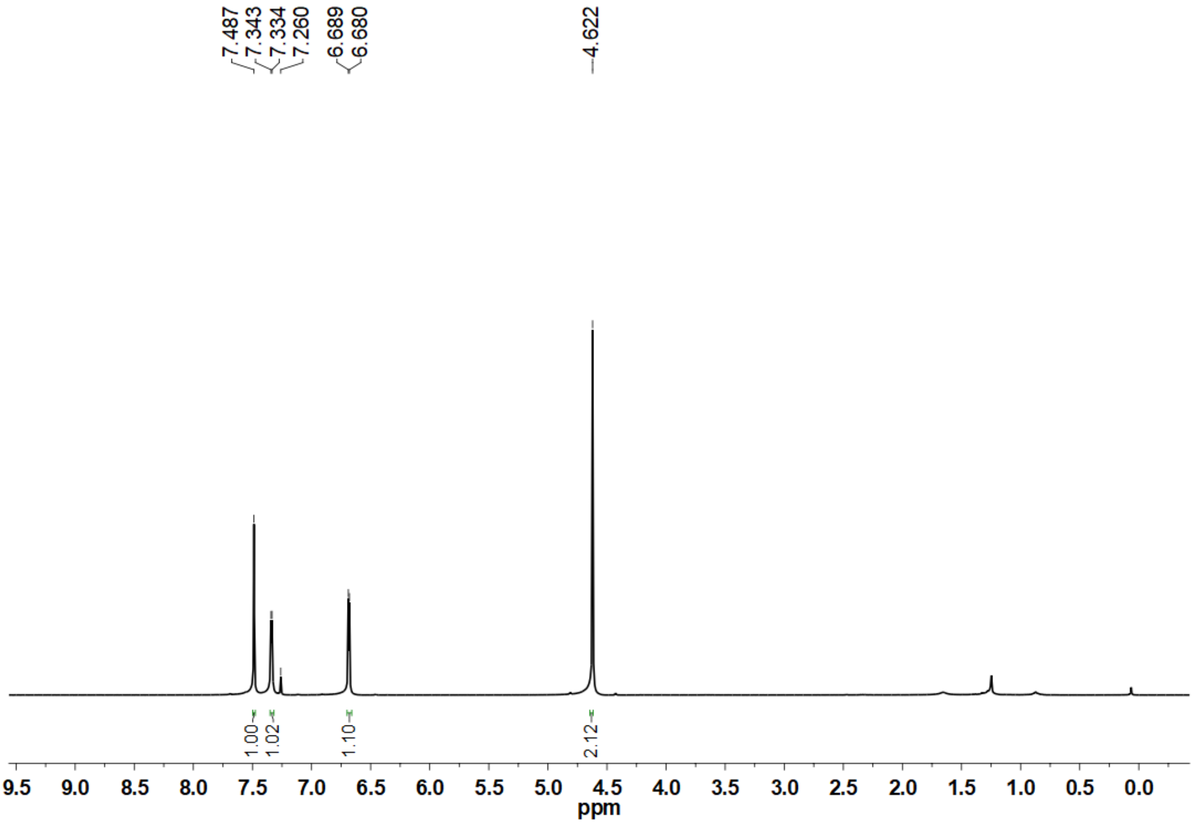

Figure S14. The ^{1}H-NMR spectrum of **3e**.

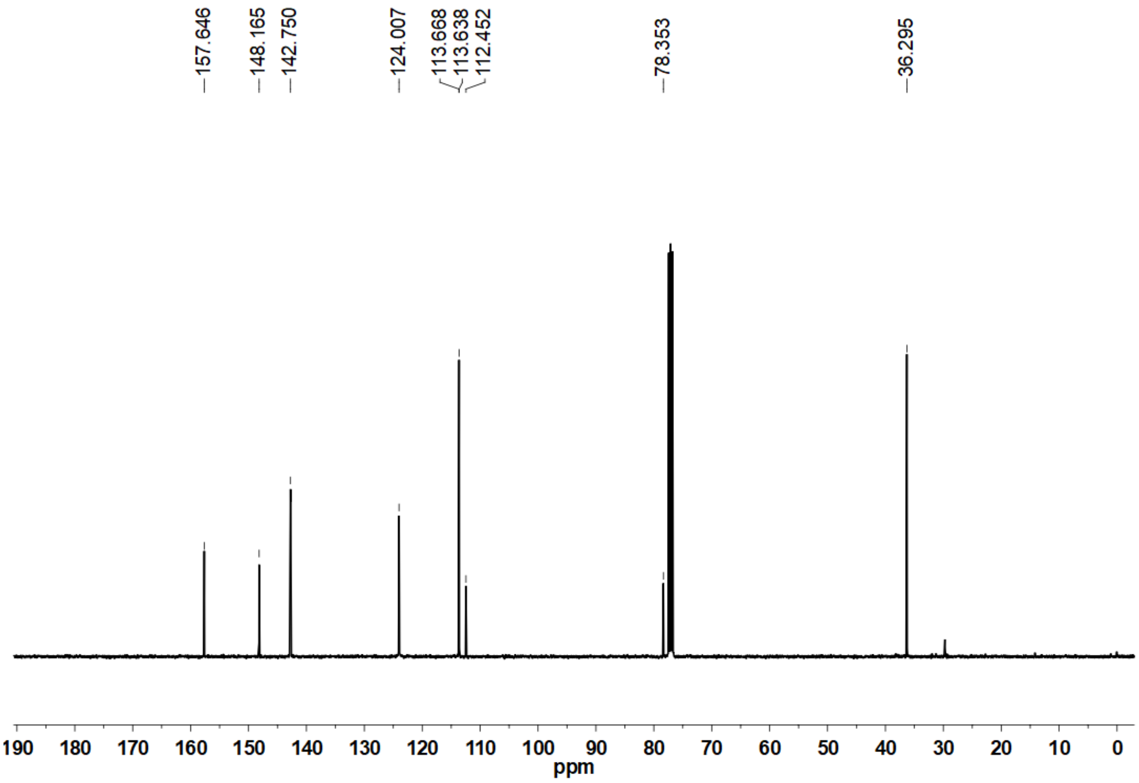

Figure S15. The ^{13}C-NMR spectrum of **3e**.

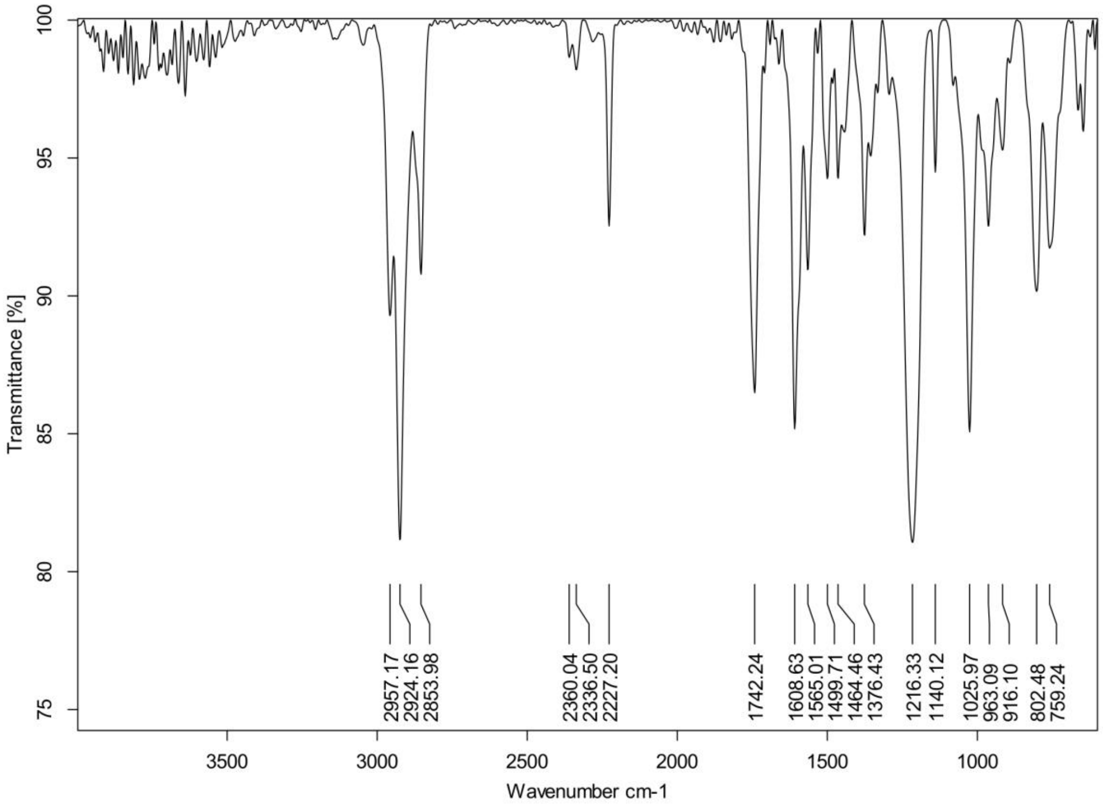

Figure S16. The FTIR spectrum of **3f.**

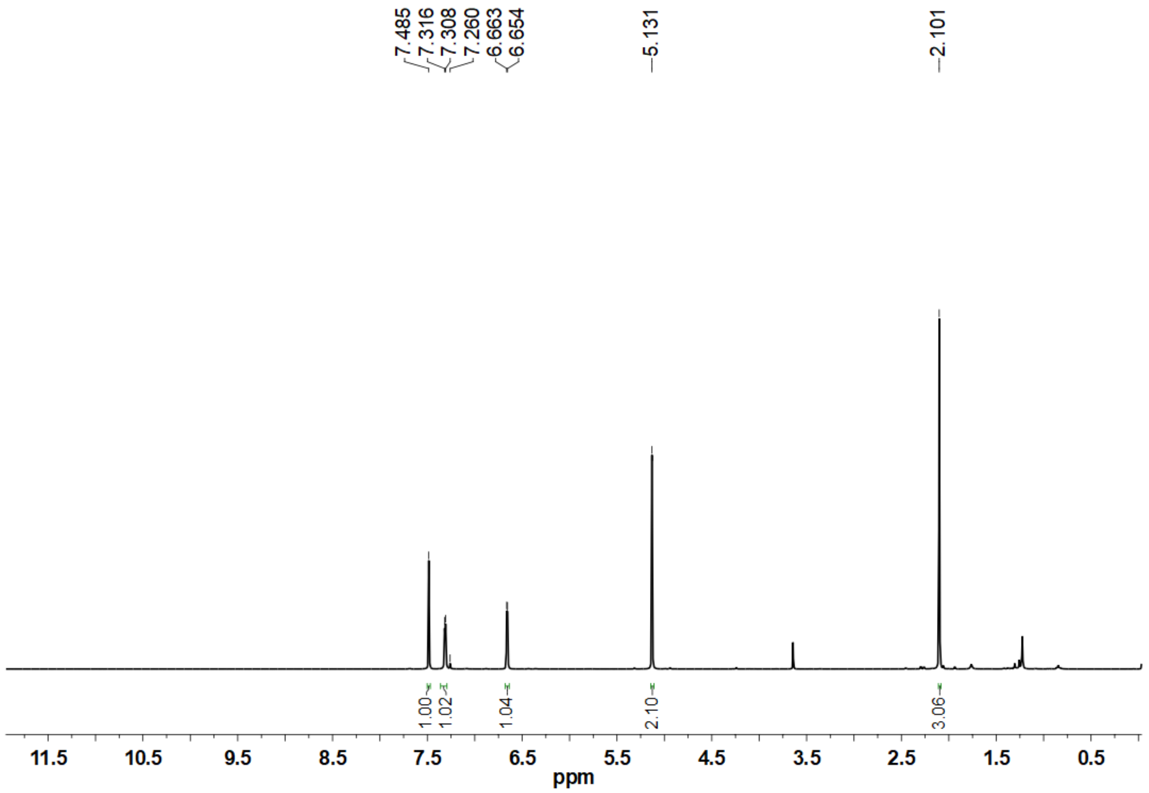

Figure S17. The ^{1}H-NMR spectrum of **3f**.

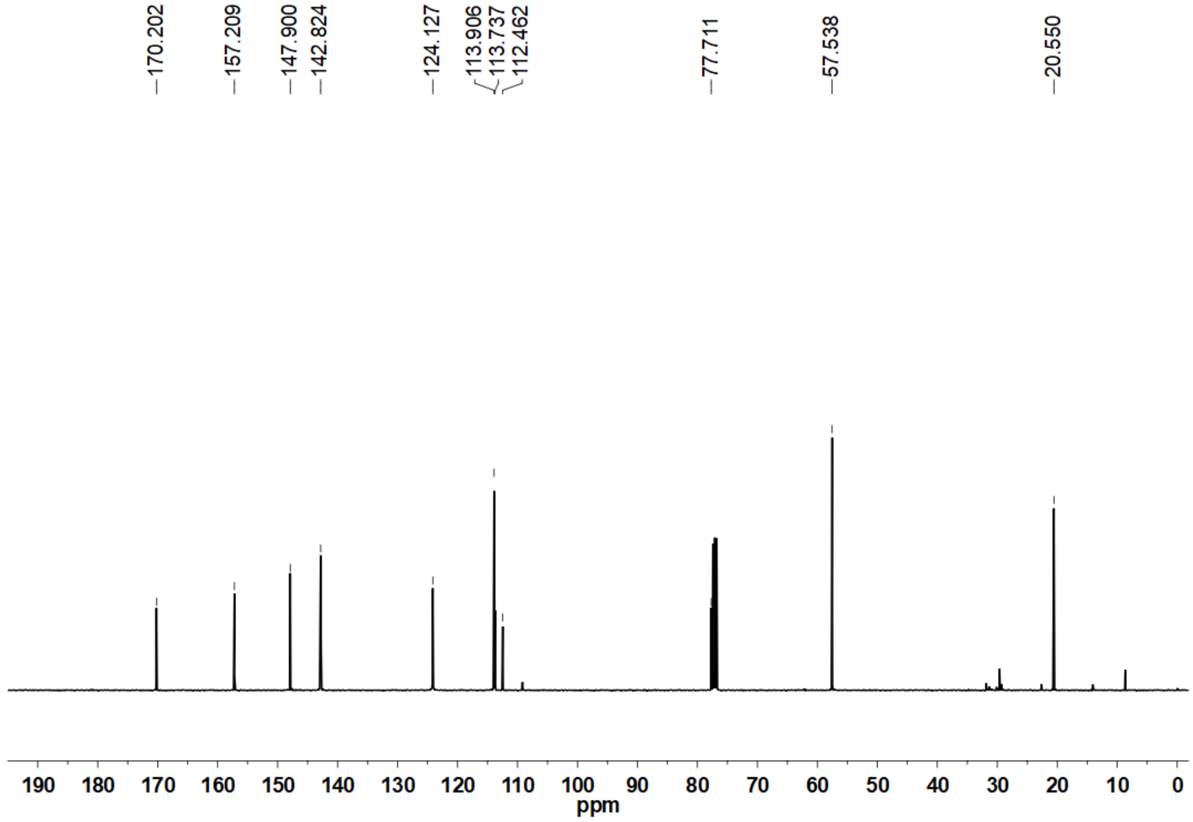

Figure S18. The ^{13}C-NMR spectrum of **3f**.

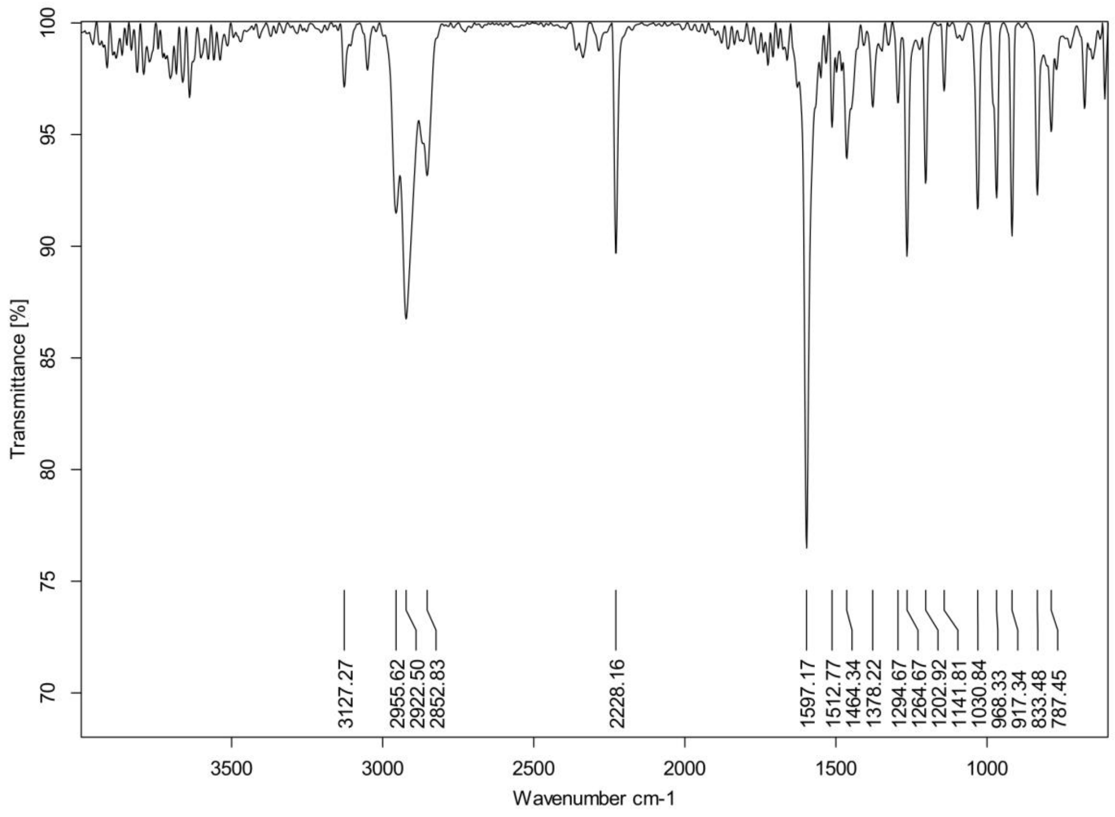

Figure S19. The FTIR spectrum of **3g.**

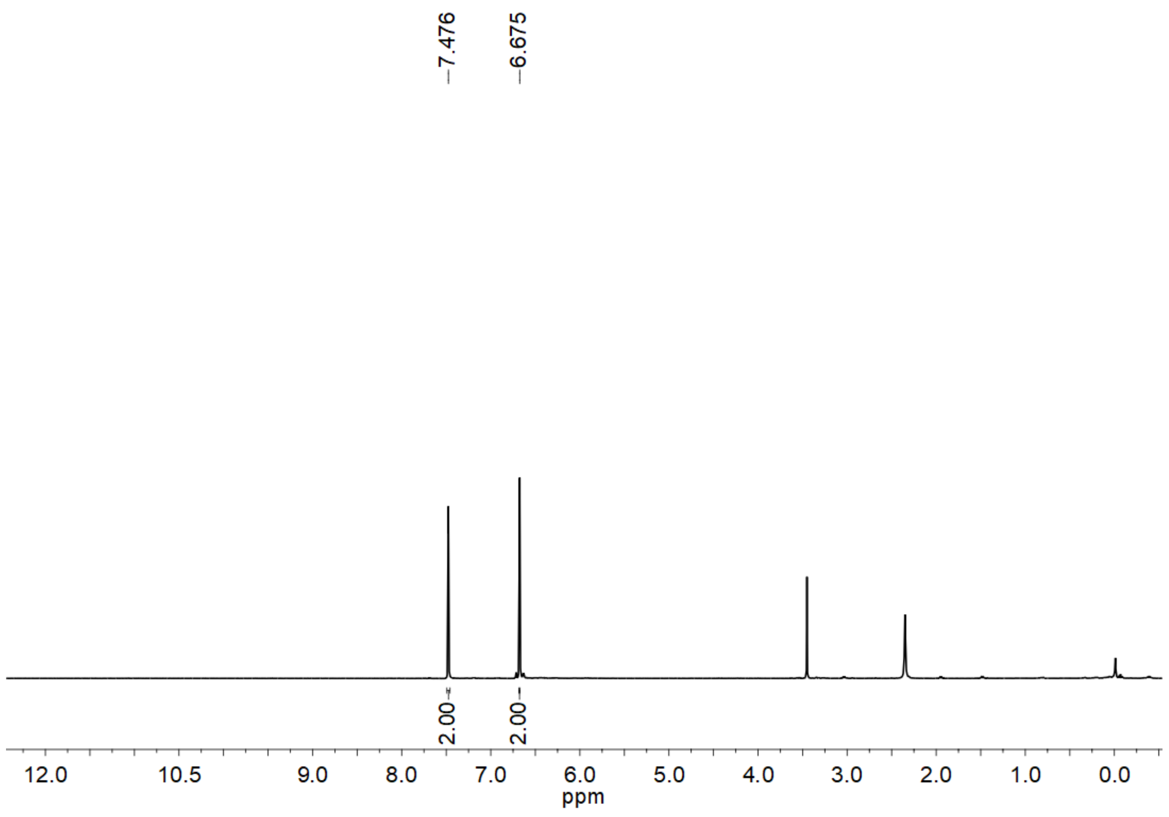

Figure S20. The ^{1}H-NMR spectrum of **3g**.

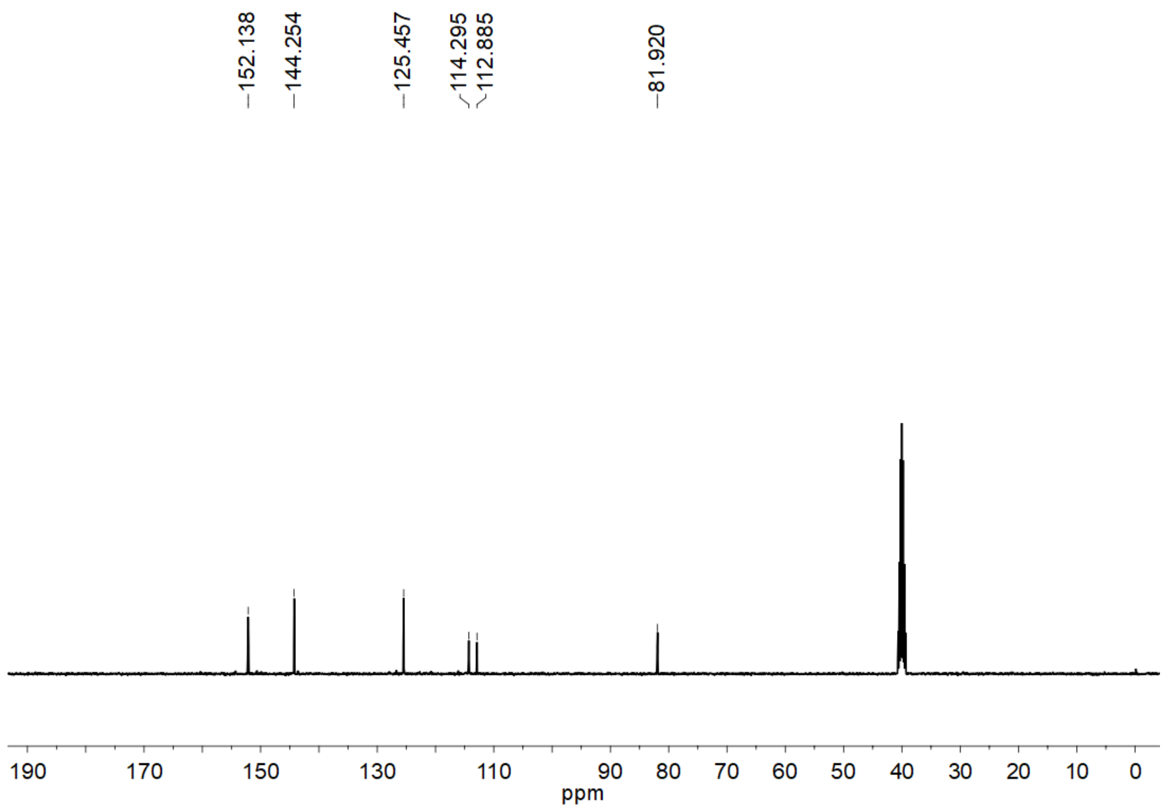

Figure S21. The ^{13}C-NMR spectrum of **3g**.

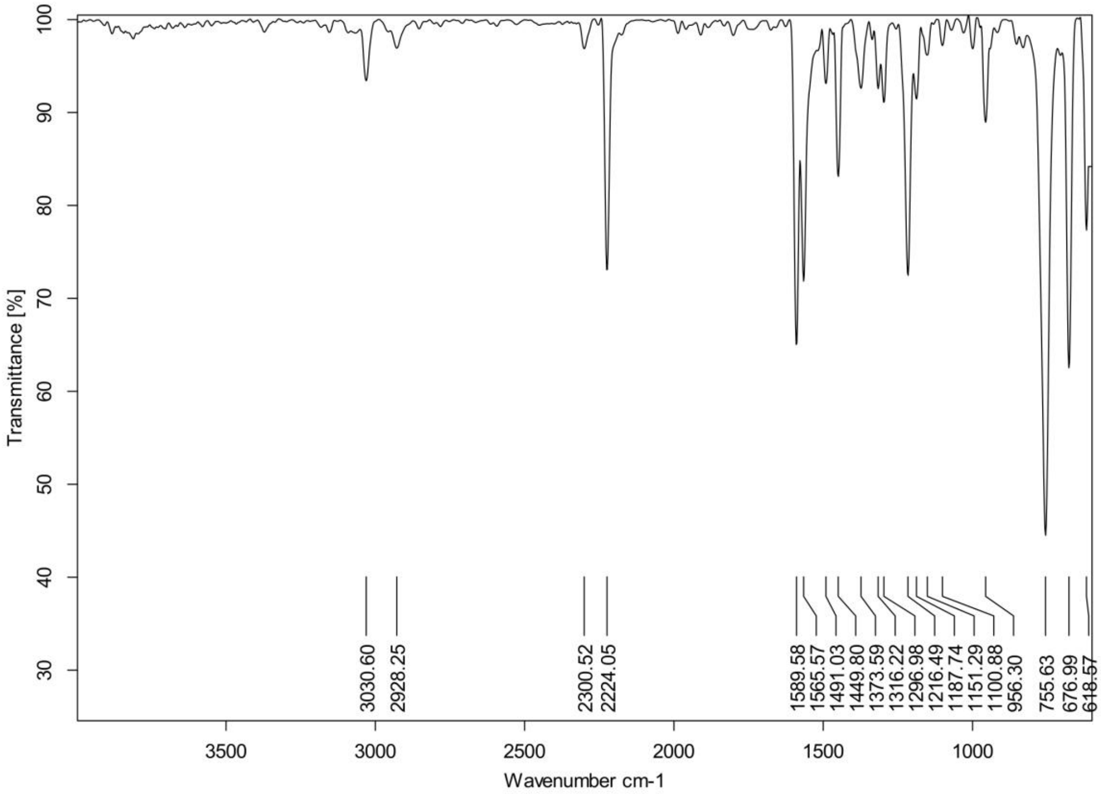

Figure S22. The FTIR spectrum of **3h.**

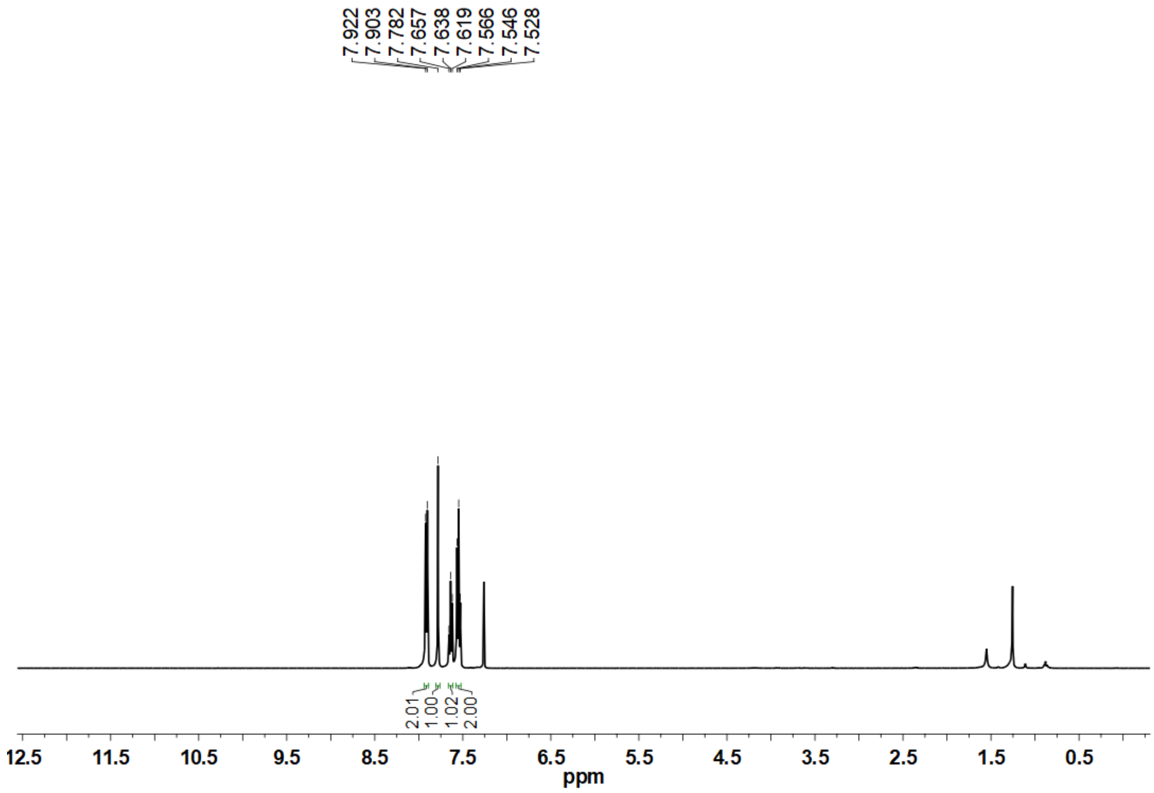

Figure S23. The ^{1}H-NMR spectrum of **3h**.

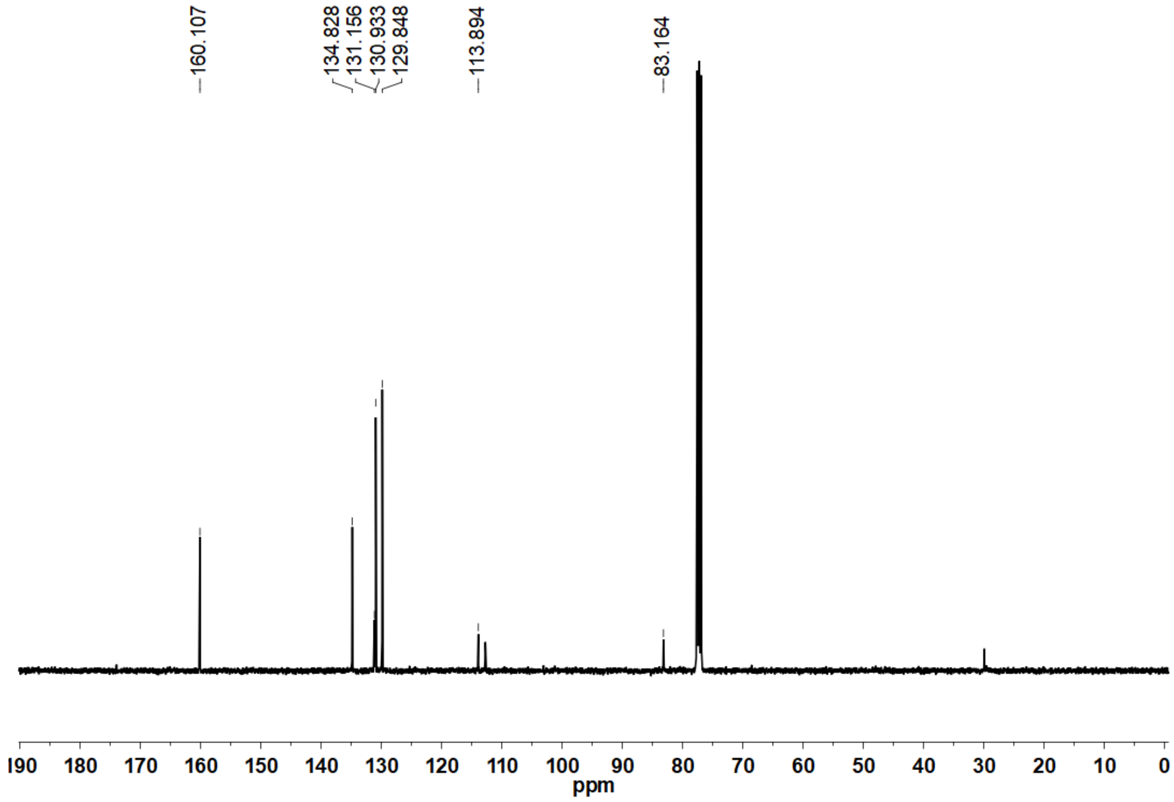

Figure S24. The ^{13}C-NMR spectrum of **3h**.

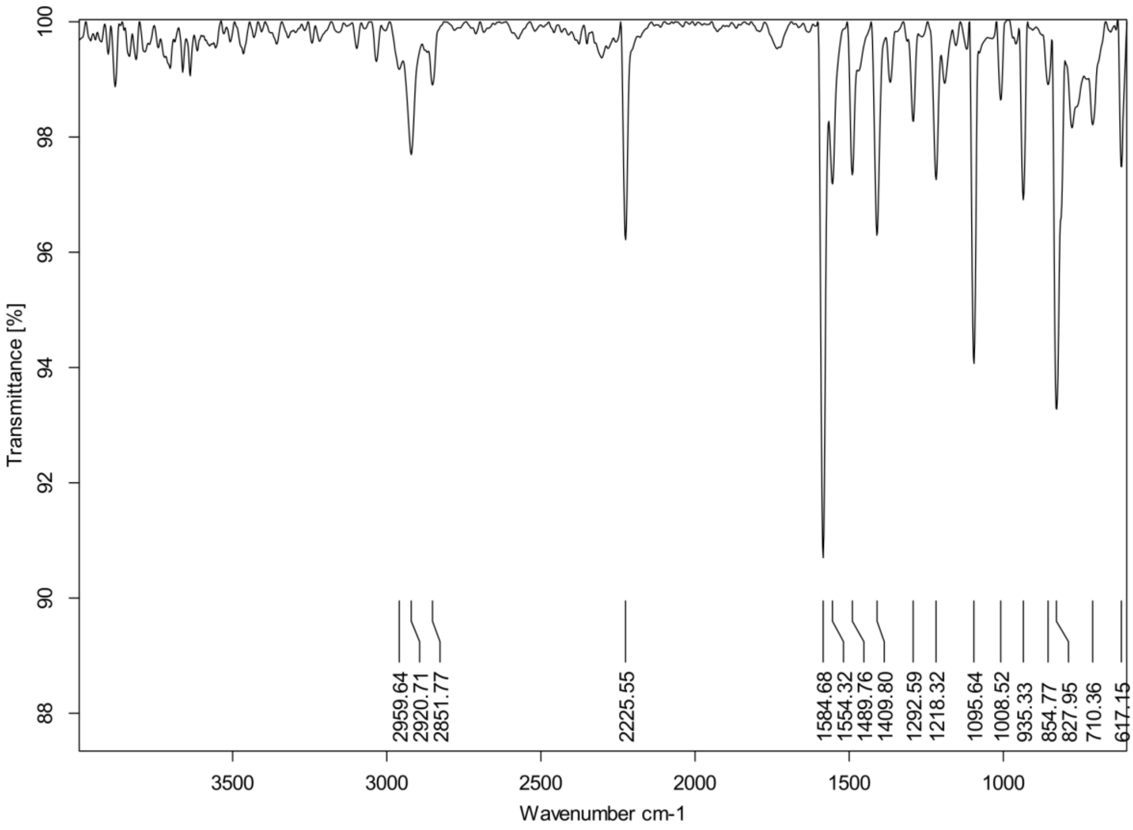

Figure S25. The FTIR spectrum of **3i**.

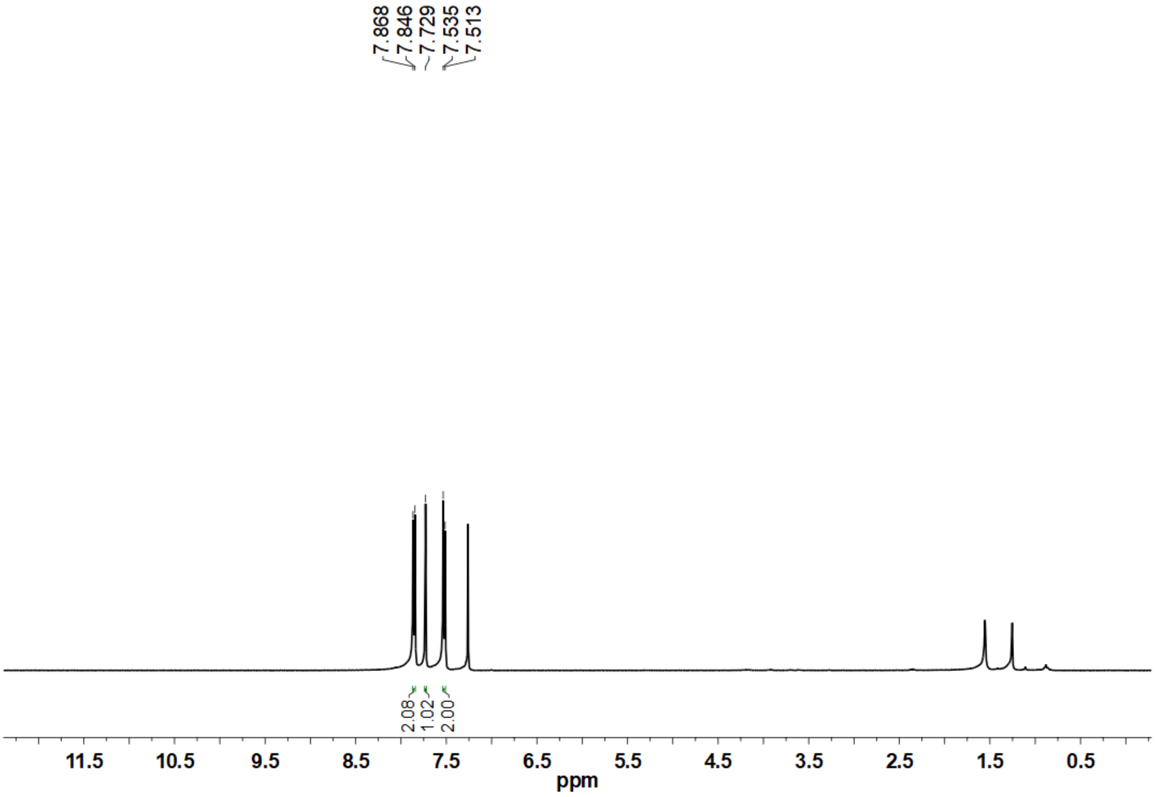

Figure S26. The ^{1}H-NMR spectrum of **3i**.

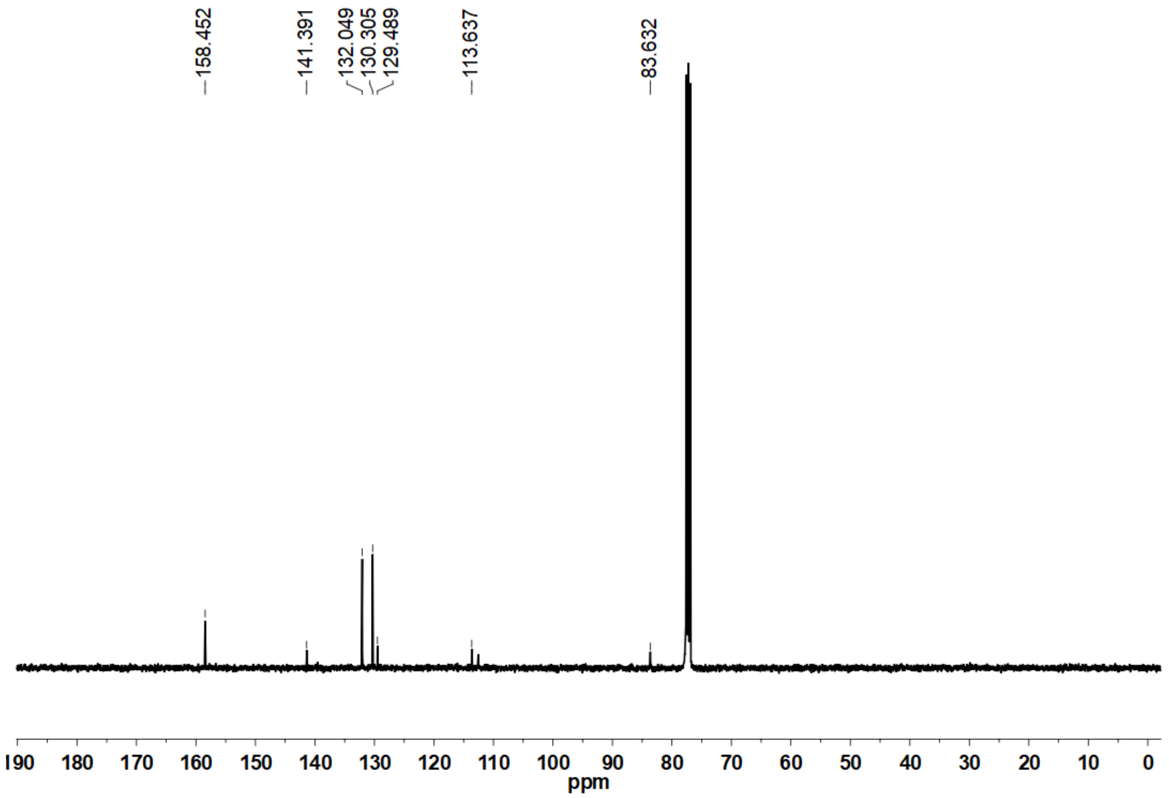

Figure S27. The ^{13}C-NMR spectrum of **3i**.

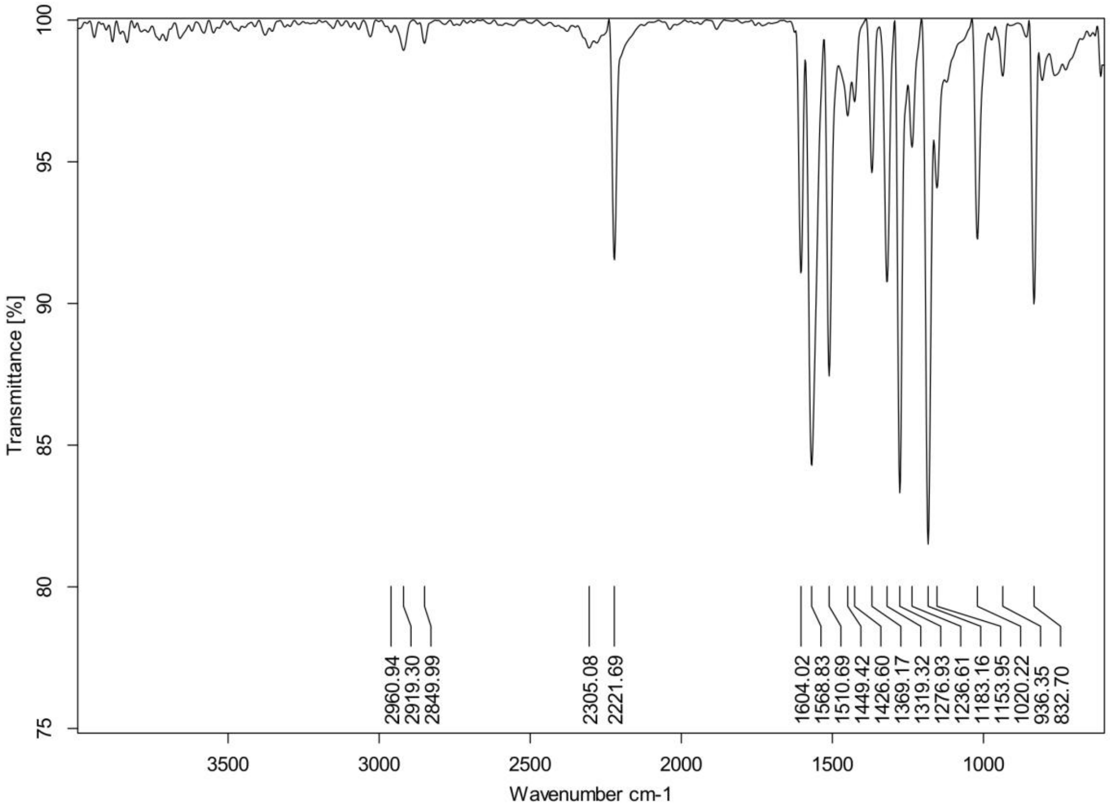

Figure S28. The FTIR spectrum of **3j**.

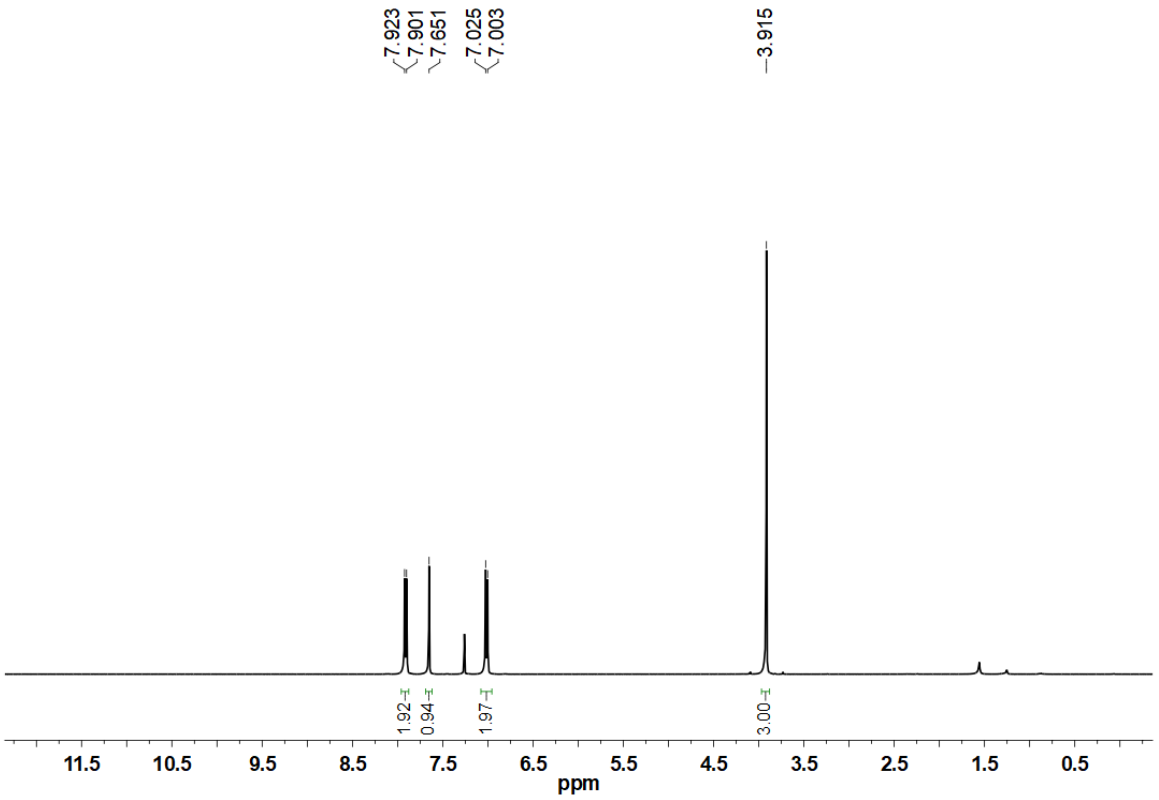

Figure S29. The ^{1}H-NMR spectrum of **3j**.

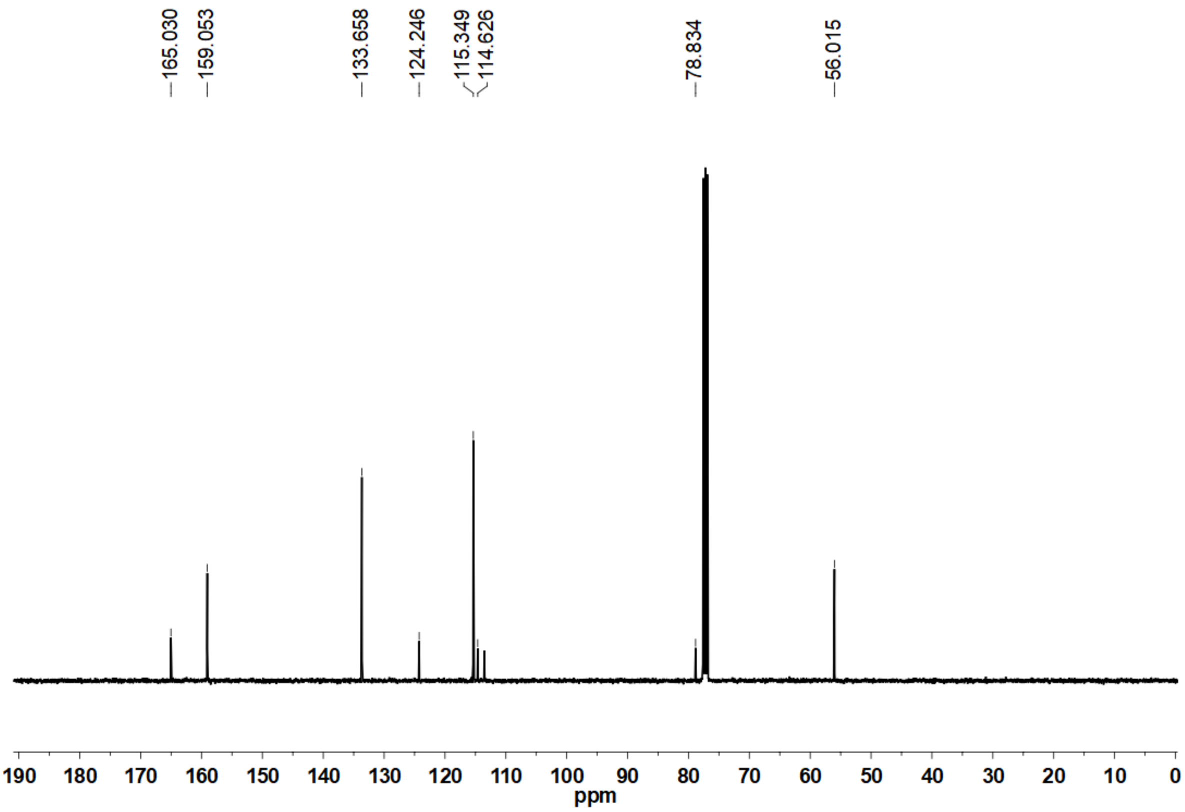

Figure S30. The ^{13}C-NMR spectrum of **3j**.

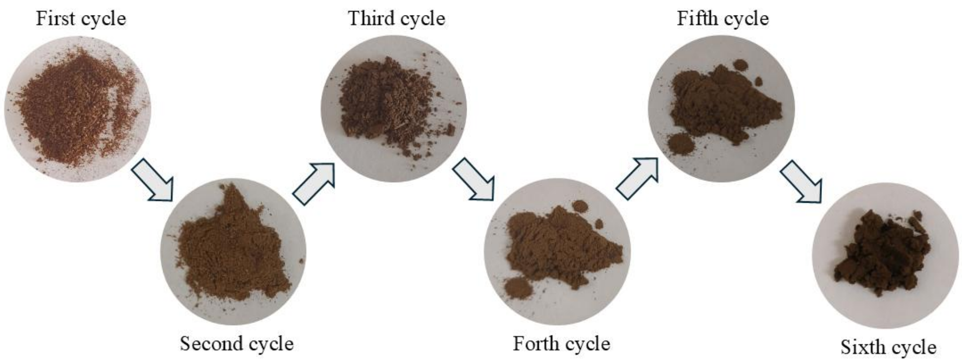

Figure S31. Visual appearance of the chitosan catalyst after each recycling step.

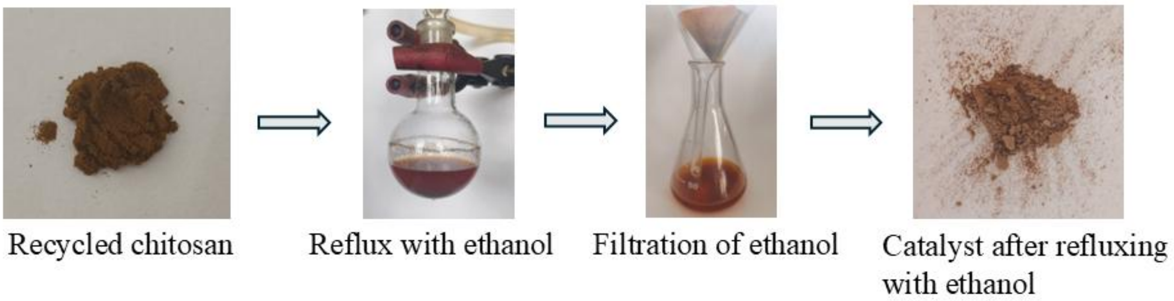

Figure S32. Attempt for removing the absorbed colored impurity from the recycled chitosan catalyst.

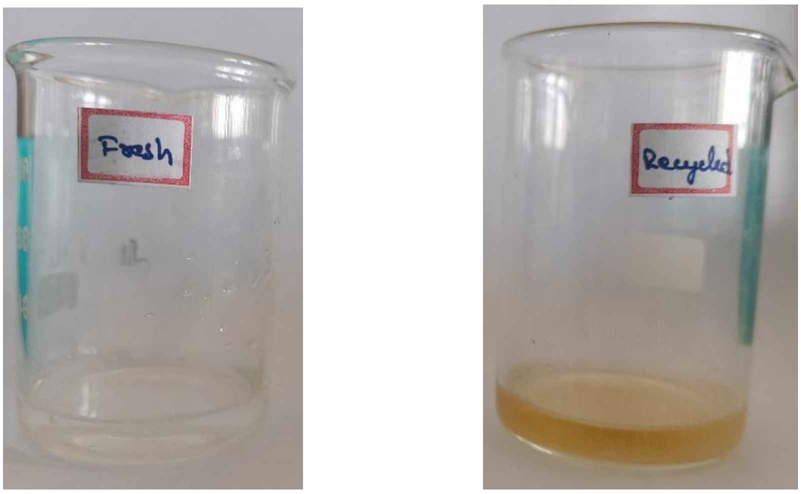

Figure S33. Fresh chitosan (left) and recycled chitosan (right) catalyst dissolved in 2% glacial acetic acid in water.

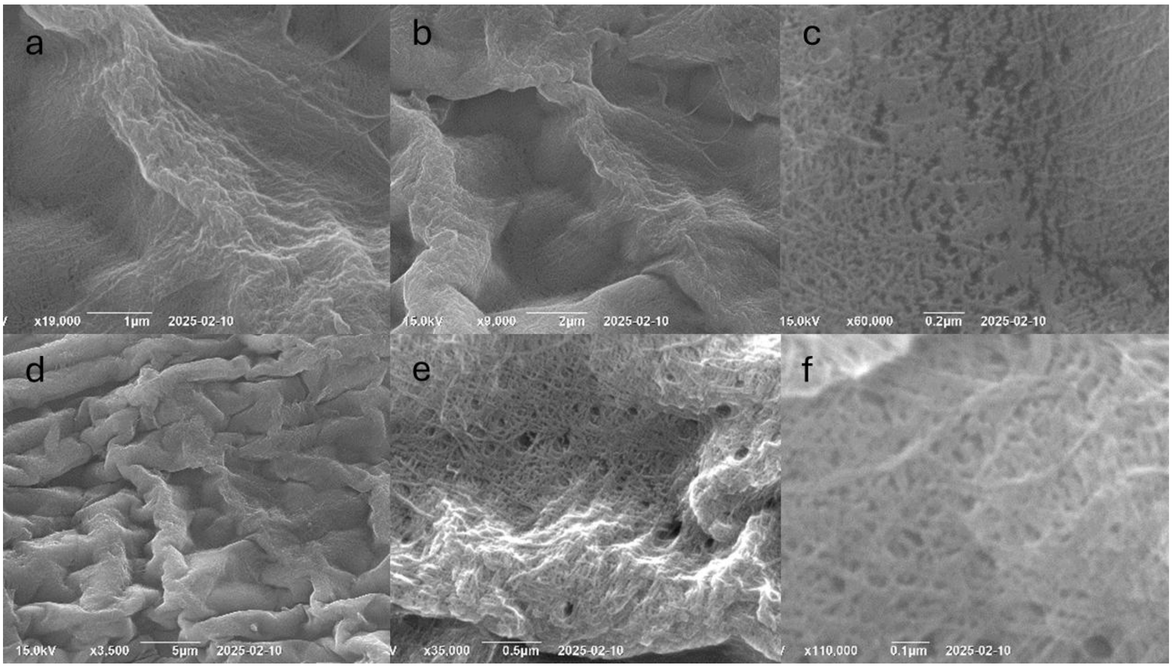

Figure S34. FESEM images of chitin at the magnification of: a) 1 μm, b) 2 μm, c) 0.2 μm, d) 5 μm, e) 0.5 μm, f) 0.1 μm.

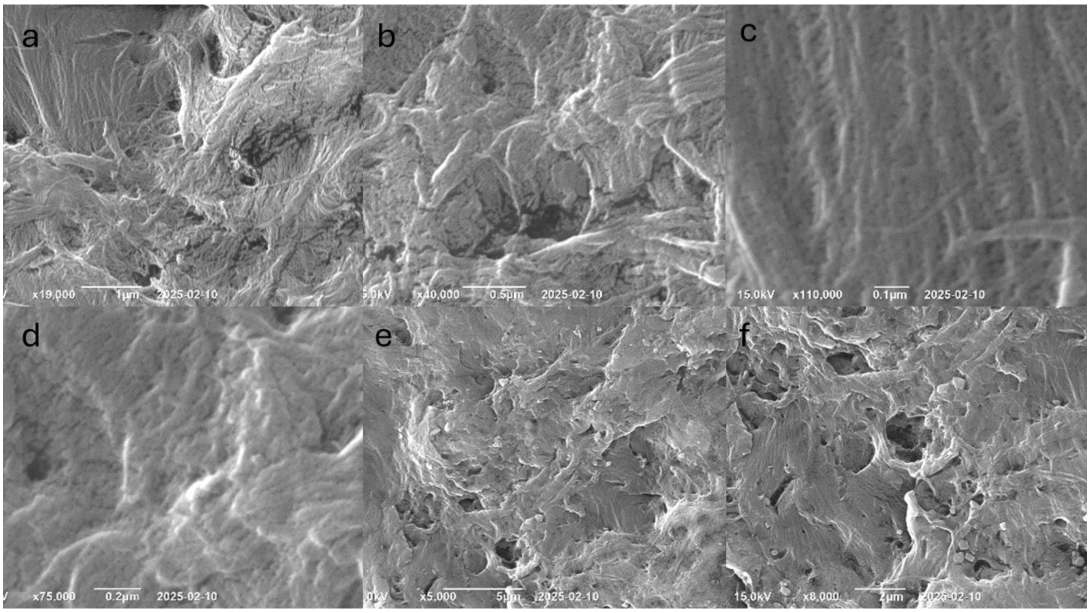

Figure S35. FESEM images of fresh chitosan at the magnification of: a) 1 μm, b) 0.5 μm, c) 0.1 μm, d) 0.2 μm, e) 5 μm, f) 2 μm.

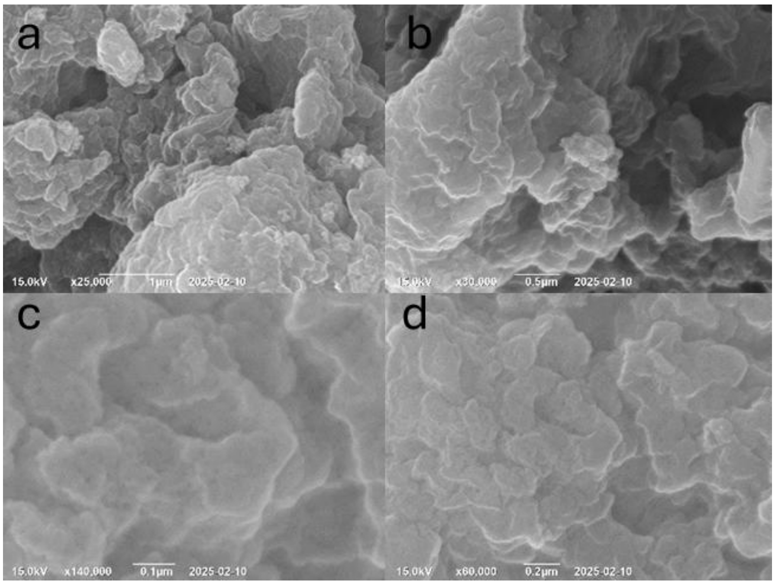

Figure S36. FESEM images of the recycled chitosan (6th cycle) at the magnification of: a) 1 μm, b) 0.5 μm c) 0.1 μm, d) 0.2μm

References

[1] Prabhakar, P. S.; Seikh, A. H.; Karim, M. R.; Dutta, S. Extending the Carbon Chain Length of Carbohydrate-Derived 5-Substituted-2-Furaldehydes by Condensing with Active Methylene Compounds under Organocatalytic Conditions. *ACS Omega* **2024**, *9* (37), 38648–38657. https://doi.org/10.1021/acsomega.4c04261.
